# Dietary Contamination with a Neonicotinoid (Clothianidin) Gradient Triggers Specific Dysbiosis Signatures of Microbiota Activity along the Honeybee (*Apis mellifera*) Digestive Tract

**DOI:** 10.3390/microorganisms9112283

**Published:** 2021-11-02

**Authors:** Sarah El Khoury, Jeff Gauthier, Sidki Bouslama, Bachar Cheaib, Pierre Giovenazzo, Nicolas Derome

**Affiliations:** 1 Department of Biology, Laval University, Québec, QC G1V 0A6, Canada; sarah.el-khoury.1@ulaval.ca (S.E.K.); jeff.gauthier.1@ulaval.ca (J.G.); sidki.bouslama.1@ulaval.ca (S.B.); pierre.giovenazzo@bio.ulaval.ca (P.G.); 2 Institut de Biologie Intégrative et des Systèmes (IBIS), Laval University, Québec, QC G1V 0A6, Canada; 3 Institute of Biodiversity, Animal Health & Comparative Medicine, University of Glasgow, Glasgow G12 8QQ, UK; bachar.cheaib@glasgow.ac.uk

**Keywords:** honeybee, clothianidin, microbiota, dysbiosis, network analysis

## Abstract

Pesticides are increasing honeybee (*Apis mellifera*) death rates globally. Clothianidin neonicotinoid appears to impair the microbe–immunity axis. We conducted cage experiments on newly emerged bees that were 4–6 days old and used a 16S rRNA metataxonomic approach to measure the impact of three sublethal clothianidin concentrations (0.1, 1 and 10 ppb) on survival, sucrose syrup consumption and gut microbiota community structure. Exposure to clothianidin significantly increased mortality in the three concentrations compared to controls. Interestingly, the lowest clothianidin concentration was associated with the highest mortality, and the medium concentration with the highest food intake. Exposure to clothianidin induced significant variation in the taxonomic distribution of gut microbiota activity. Co-abundance network analysis revealed local dysbiosis signatures specific to each gut section (midgut, ileum and rectum) were driven by specific taxa. Our findings confirm that exposure to clothianidin triggers a reshuffling of beneficial strains and/or potentially pathogenic taxa within the gut, suggesting a honeybee’s symbiotic defense systems’ disruption, such as resistance to microbial colonization. This study highlights the role of weak transcriptional activity taxa in maintaining a stable honeybee gut microbiota. Finally, the early detection of gut dysbiosis in honeybees is a promising biomarker in hive management for assessing the impact exposure to sublethal xenobiotics.

## 1. Introduction

Honeybees are important pollinators that benefit nature and agriculture [1]. However, they are continuously in contact with chemical agents, e.g., neonicotinoids [2], which are intensively used for crop protection against vector enemies [3]. Because neonicotinoids are soluble in water, honeybees are by consequence directly in contact with these stressors via the water intake [4]. Furthermore, due to their persistence in the environment [5,6], especially in pollen [4], neonicotinoids have been found both on the honeybee body [7] and internally, e.g., within gut cells [8]. The resulting bioaccumulation has been shown to alter honeybee physiology [9,10], cognition [11], neuronal communication [12,13], and immunity [14]. Given that gut microbes are of primary importance in regulating the above-mentioned beneficial functions in honeybees [15,16,17,18], an increasing number of studies have focused on understanding the relationship between symbiotic microbes and bee health [15]. Recent research observed that exposure to neonicotinoids (thiacloprid [19], nitenpyram [20] and imidacloprid [21]) exerted adverse effects on gut microbiota homeostasis. Clothianidin (CAS 210880-92-5) was shown to impact microbiota structure and has been suspected of potentially disrupting the microbiota–immunity axis [21,22,23]. Thus, there is an urgent need to understand to what extent those toxic compounds impact the homeostasis of the honeybee gut microbiota. Recent studies on gut microbiota have evidenced its involvement in crucial health-related functions such as detoxification [24], host immunity [15] and pathogen prevention [25]. Control of innate immunity by the gut microbiota was found in Drosophila, through the *NF-κB* pathway [26]. In honeybees, the *NF-κB* pathway expression was observed to decrease following exposure to sublethal doses of clothianidin (0.1 to 10 ppb (μg/L)) [22]. Given the importance of the gut microbiota on the honeybee immunity [18,27], we expect that the microbiota–immunity axis is disrupted by sublethal clothianidin concentrations. 

Anatomically, the honeybee gut is partitioned into four distinct sections: crop, midgut, ileum and rectum. Characterized by low taxonomic diversity [28], a healthy honeybee gut microbiome harbors between five and nine phylotypes, representing more than 98% of the bacterial 16S rRNA gene sequences [29] and subdivided into core members (i.e., present in almost all individuals), non-core members [30] and low abundance taxa, which are thought to play an important role in a microbial community [31]. The reappearance of particular honeybee gut microbiota in non-culture-based 16S rDNA and metagenomic sequences datasets suggests that most prevalent microorganisms in the gut microbiota were inherited from hive members and not acquired from the external environment: *Lactobacillus* (Firm-5, Firm-4), *Bifidobacterium* spp., *Gilliamella apicola* and *Snodgrassella alvi* are ubiquitous in honeybees; whereas the presence of *Frischella perrara*, *Bartonella apis*, *Apibacter adventoris* and/or *Parasaccharibacter apium* is more variable [18,30,32,33]. Acquisition of each honeybee’s distinct gut microbiota occurs by trophallaxis between newly emerged bees and nurse bees [34]. 

Pesticides may have both negative and positive effects on microbial communities [35,36]. Pesticide metabolization is a source of nutrients for some tolerant microbial strains, and harms others, possibly killing more vulnerable ones [37,38]. Such ecological changes within the microbial community structure can set off various chain reactions, depending on the tolerance of the microbial strain reacting to the pesticide first. For example, pesticide-tolerant microbes can benefit from the removal or reduction of pesticide-sensitive microbes and thrive under conditions of less competition for resources [39]. In previous studies investigating microbiota response to clothianidin, field exposure of bumblebees was reported to only induce marginal shifts in gut microbiota [40], whereas no significant changes were observed in soil microbial structure [41,42]. Conversely, other studies reported clothianidin degradation by microbial [43,44,45] and photocatalytic activities [46]. Furthermore, depending on their metabolism, microorganisms are able to degrade neonicotinoids into less or more toxic metabolites, thereby controlling their impact on environmental integrity [47] and possibly on the network of interacting microbiota. Therefore, the degree to which the microbiota–immunity axis is disturbed depends on the ability of the gut microbiota to metabolize pesticides. For instance, *Drosophila melanogaster* gut microbiota degrades chlorpyrifos [48], while *Apis mellifera* gut microbiota cannot metabolize imidacloprid [49]. Degradation can differ depending on the chemical agent [43,50], the microbial strains involved [43,50], and the metabolites generated, which may be more toxic than the parent molecule [51]. Thus, we hypothesized that clothianidin induces gut microbiota dysbiosis in honeybees, and we aimed to investigate to what extent the different sections of the gut microbiota respond specifically to sublethal clothianidin concentrations tested in previous studies, ranging from 0.1 to 10 ppb [11,22]. Because of the importance of stable positive correlations between gut microbiota members [52] for limiting colonization by pathogens [53], we monitored the dynamics of negative/positive correlations between gut microbial symbionts, as markers of microbiota dysbiosis [54,55,56,57]. 

In this study, we implemented a reverse-transcribed 16S rRNA-based metataxonomic approach to characterize the gut microbiota’s functional dynamics. The 16S rRNA metataxonomic approach (i.e., quantifying the taxa’s overall gene expression with their relative 16S rRNA transcript copy numbers) allows for an evaluation of the functionally active taxonomic diversity and the relative contribution of each bacterial strain to the overall microbiota activity, thereby providing relevant insights for deciphering their functional roles [58]. The 16S rRNA transcript expression levels per taxon (hereby called activity) were used to construct co-expression networks in order to detect and quantify community activity changes during gut microbiota dysbiosis [59]. Furthermore, we evaluated to what extent the alpha diversity and monitoring ratio of positive/negative correlations in bacterial taxonomic networks are an effective approach for detecting and/or quantifying the dysbiosis process when the host organism is facing stress. Most studies to date have targeted one microbial niche (i.e., one type of host tissue) and one level of stress factor [54,60] or a combination of individuals with various levels of a given factor [55,61], with most of these using 16S rDNA metataxonomics. To obtain a more comprehensive assessment of the gut microbiota response to exposure to clothianidin, we targeted three gut sections, which are differentiated in terms of microbiota functional activity [30]. This study aims to provide further insights into the correlations between the host, its microbiota and exposure to xenobiotics.

## 2. Materials and Methods

### 2.1. Experimental Setup

Cage experiments on honeybees were conducted in the middle of July and August 2017 at the Centre de Recherche en Sciences Animales de Deschambault (CRSAD, Deschambault, QC, Canada). All bees used for this study originated from two European honeybee colonies (*Apis mellifera* L.) headed by sister queens. Newly emerging bees were obtained as described in Williams et al. [62] using a “nursery colony” made of a Langstroth hive body with five combs of capped brood (purple eye), one frame of honey and pollen, and some adherent nurse bees (approximately 20–30 nurse bees per frame) from the original colonies. The nursery colony was incubated at 32 °C and 55% relative humidity in a model 3040 apparatus (Forma Scientific Inc., Marietta, OH, USA) for six days. Young bees emerged in nursery colonies and were kept there for 4–6 days to ensure optimal microbiota acquisition/colonization [34]. After this incubation period, these young bees were hand collected and placed in cages. Each cage consisted of a Plexiglas structure (10 × 10 × 10 cm) adapted from [63] with an inverted sterile syringe (20 mL, BD, Franklin Lakes, NJ, USA) containing 50%_w/v_ sucrose syrup (sugar diluted in distilled water). Bees rapidly learned to take the syrup from the bottom opening of the syringe. Then, 200 bees were randomly distributed in each cage (5 cages per group) for a total of 4000 bees. Cages were kept in a controlled environment room (32 °C ± 1 °C and 50% ± 5% relative humidity) for 28 days. Cages were randomly distributed between groups and clothianidin administration began on day 3 (7–9 days post-emergence). Each exposed group was supplied with 50%_w/v_ sucrose solution supplemented with the tested clothianidin concentration. Experimental groups were defined as follows: three clothianidin concentrations (0.1, 1 and 10 ppb) and a control group (50%_w/v_ sucrose solution without clothianidin). Mortality was recorded daily in each cage. Once a week (T = 7, 14, 21 and 28), 20 bees were sampled from each cage and stored at −80 °C.

### 2.2. Neonicotinoid Preparation and Quantification

Clothianidin (CAS Number 210880-92-5) was supplied by Sigma-Aldrich, Inc. (Oakville, ON, Canada) and dissolved in distilled water to obtain three different final concentrations (0.1, 1 and 10 ppb). Clothianidin was quantified by liquid chromatography–tandem mass spectrometry (LC-MS/MS) at the INRS (Institut National de la Recherche Scientifique, Québec, QC, Canada). Clothianidin concentration in honeybees was measured with a modified QuEChERS method adapted from Paradis et al. [64] For each experimental condition, 10 individual honeybees were sampled and pooled for LC-MS/MS analysis (in triplicates). Prior to honeybee gut dissection and pooling for clothianidin quantification experiments with LC-MS/MS, honeybees were washed using a diluted bleach solution (1:100) for 2 min to ensure all clothianidin residue was removed from the surface of the body. Then, each honeybee was rinsed separately three times in clean distilled water to remove bleach residues. This was followed by centrifugation for 45 s at 10,000 g at 20 °C to remove all remaining residue. Stock standards for use in calibrating and determining recovery were formulated with methanol (MeOH) and stored at 4 °C in a dark room. The internal standard (IS) atrazine-D5 was purchased from CDN Isotopes (Pointe-Claire, QC, Canada). Samples were mixed with acetonitrile (CH_3_CN—1.5 mL) and vortexed for 2 min. For extraction, a mixture of salts was added to each sample: magnesium sulfate (MgSO_4_—4 g), sodium chloride (NaCl—1 g), sodium citrate dihydrate (1 g) and disodium citrate sesquihydrate (0.5 g). Then, each mixture was agitated up and down for 15 min, and centrifuged at room temperature for 5 min at 3000 g. The mixture was decanted, and the supernatant (500 μL) was transferred to a new culture tube. The mixtures were then dehydrated in a nitrogen evaporator set at 40 °C. Then, 100 μL of water:methanol (85:15) + atrazine-D5 (2 ppb) was added to dry pellets, and 100 μL of the resulting solution was transferred to a new tube for pesticide quantification using LC-MS/MS. 

### 2.3. Chromatography and Quantitative Analyses 

Liquid chromatography analyses were carried out using a TSQ Quantum™ Access MAX Triple Quadrupole Mass Spectrometer (Thermo Scientific, San Jose, CA, USA) equipped with a column: Hypersil Gold aQ (Thermo Scientific) 5 μm, 2.1 × 100 mm at T = 40 °C. The column temperature and chromatographic gradient were optimized to avoid tailing peaks and to improve peak shapes. A gradient system was applied to achieve the best separation of clothianidin molecules. Chromatographic separation was performed at 40 °C with an injected volume of 10 μL and a run time of 18 min. The mobile phase consisted of a mixture of 100% 10 mM Acetate Ammonium:Methanol (85:15) for 1 min. The flow rate was set at 0.25 mL/minutes. Clothianidin was separated with the following elution program: linear gradient from 1.1 to 3 min (15% Methanol: 85% 10 mM Acetate Ammonium to 85% Methanol: 15% 10 mM Acetate Ammonium, respectively), and return to the initial conditions—100% 10 mM Acetate Ammonium: Methanol (85:15) for 10 min. 

Ionization was performed by an electrospray source (ESI) in positive ionization in SRM mode, the ion tube was heated to 350 °C and the ions were detected by triple quadrupole mass spectrometer: Clothianidin was as follows: *m*/*z* 250 (precursor ion), *m*/*z* 169 (product ion), Atrazine-D5 *m*/*z* 221 (precursor ion) and *m*/*z* 179 (product ion). Quantification was performed with Xcalibur Software (Thermo Scientific). Each substance was characterized by its retention time (RT) and a quantification transition. Quantitative analysis of clothianidin traces was determined by relating the area ratio (peak area of clothianidin/peak area of internal standard) of each sample to the calibration curve of the clothianidin standard. 

### 2.4. Feeding Rate

Every syringe was weighed daily to measure syrup consumption average per cage. Syrup consumption per honeybee was calculated as the total measured syrup consumption per cage divided by the average of the sum of living bees present at (T = t − 1) and (T = t) per cage. The normality of distribution was assessed mathematically with the Kolmogorov–Smirnov test [65] and then confirmed with the Shapiro–Wilk test [66]. For pairwise statistical tests, we used the Wilcoxon signed-rank test [67] because the data did not meet the assumption of normality. For post hoc comparisons between groups at specific time points, we used the Kruskal–Wallis test [68]. 

### 2.5. Survival Analysis 

To estimate honeybee survival rates, we used the Kaplan–Meier estimator [69] in the survival R package (version 3.2.7) [70]. Statistically significant risk differences between treatments were detected with a Cox’s proportional hazards regression using the coxph model implemented in the survival R package, as previously described [71].

### 2.6. RNA Extraction

Tissue sampling targeted three honeybee gut sections (midgut, ileum and rectum) at T = 7. For RNA extraction, samples from the same cage were pooled (5 gut sections from 5 bees per cage) and RNA was extracted using the TriReagent method (Ambion, Thermo Fisher Scientific). Intestinal tissues were placed into a 2 mL microtube containing 1 mL of TriReagent. Each sample was crushed with a sterilized grinder, and then incubated at room temperature (RT) for 5 min. Then, 200 μL of fresh chloroform per 1 mL of TriReagent was added to each sample and vortexed for 15 s. Samples were incubated for a second time at RT for 12 min and vortexed at half-time, then centrifuged for 15 min at 12,000 g at 4 °C. Next, 400 μL of the upper aqueous phase was transferred into a new 1.5 mL microtube. Then, 250 μL of isopropanol and 250 μL of hypersaline solution (1.2 M Trisodium citrate; 0.8 M NaCl) were added per mL of TriReagent. A few inversions followed to mix the solutions together, which were incubated at RT for 10 min. Then, samples were centrifuged for 15 min at 12,000 g at 25 °C and the supernatant was removed. Next, 1 mL of 75% ethanol was added per mL of TriReagent, followed by centrifugation for 15 min at 12,000 g at 25 °C. The supernatant was discarded. The RNA pellet was then air-dried and dissolved in 30 μL of nuclease free water. 

### 2.7. 16S rRNA Gene Sequencing 

#### 2.7.1. cDNA Synthesis

RNA samples were reverse transcribed into complementary DNA (cDNA) with the qScript^TM^ cDNA SuperMix method (QuantaBio, VWR, Beverly, MA, USA) by following the manufacturer’s protocol [72]. Then, partial 16S rDNA amplicons of the hypervariable V3-V4 regions were obtained in a two-step dual indexing procedure. 

#### 2.7.2. Two-Step 16S rDNA Amplicon Library Preparation

First, the V3-V4 hypervariable region was amplified by PCR using universal primers (10 µM) [347-F (5′-ACACTCTTTCCCTACACGACGCTCTTCCGATCT-GGAGGCAGCAGTRRGGAAT-3′) and 803-R (5′-GTGACTGGAGTTCAGACGTGTGCTCTTCCGATCT-CTACCRGGGTATCTAATCC-3′)] (Sigma-Aldrich Life Science, Oakville, ON, Canada), which were tailed on the 5′ end with part of the Illumina TruSeq adapters. The first PCR was conducted in a total volume of 50 µL: reaction buffer (Q5) (5×) 10 µL; dNTPs (10 mM) 1 µL; 347-F 2.5 µL; 803-R 2.5µL; high GC enhancer (Q5) (5×) (NEB) 10 µL; Q5 high-fidelity DNA polymerase (NEB) 1 µL; H20 20 µL; and DNA template 3µL. After initial denaturation at 98 °C for 2 min, amplification was performed using 35 cycles of 10 s at 98 °C, 30 s at 60 °C and 30 s at 72 °C followed by a final extension at 72 °C for 2 min. PCR reactions were purified using the sparQ PureMag Beads PCR cleanup kit (Quantabio, VWR, Ontario, Canada). Amplification products were run on 2.0% agarose gels to detect fragments of expected size (~450 bp), then quantified using a Nanodrop 1000 spectrophotometer (Thermo Fisher, Ottawa, ON, Canada).

Then, a second PCR was performed to attach the remaining adapter sequences (regions that anneal to the flow cell and library specific barcodes). The second PCR was conducted in a total volume of 50 µL: reaction buffer (Q5) (5×) 10 µL; dNTPs (10 mM) 1 µL; generic forward primer (5′-AATGATACGGCGACCACCGAGATCTACAC-[index1]-ACACTCTTTCCCTACACGAC-3′) (Sigma-Aldrich) (10 µM) 1 µL; reverse primer (5′-CAAGCAGAAGACGGCATACGAGAT-[index2]-GTGACTGGAGTTCAGACGTGT-3′) (Sigma-Aldrich) (10 µM) 1 µL; high GC enhancer (Q5) (5×) (NEB) 10 µL; Q5 high-fidelity DNA polymerase (NEB) 1 µL; H20 24 µL; and DNA template (10–15 ng/µL) 3µL. After initial denaturation at 98 °C for 2 min, amplification was performed using 12 cycles of 10 s at 98 °C, 30 s at 60 °C and 30 s at 72 °C followed by a final extension at 72 °C for 2 min. The quality of purified PCR products was verified on a DNA 7500 BioAnalyzer chip (Agilent) in order to detect an insert fragment size of 550 bp, then quantified using a Nanodrop 1000 dpectrophotometer.

#### 2.7.3. Paired End Illumina Sequencing

Barcoded amplicons were pooled in equimolar concentrations and sequenced at the “Plate-forme d’Analyses Génomiques” of Laval University (Québec, Canada) using Illumina MiSeq paired end technology (2 × 300 bases). We used 15–20% of the PhiX control v3 Library (MiSeq Reagent kit v3 600 cycles PE, Illumina, Inc., San Diego, CA, USA) in the sequencing runs as a calibration control.

#### 2.7.4. Disclaimer

Please note that primers used in this work contain Illumina-specific sequences protected by intellectual property (Oligonucleotide sequences © 2021–2013 Illumina, Inc., all rights reserved. Derivative works created by Illumina customers are authorized for use with Illumina instruments and products only. All other uses are strictly prohibited). 

### 2.8. Bioinformatics Analysis

#### 2.8.1. Sequence Clustering

In total, 120 samples (2 replicates of 5 bees × 3 gut sections × 5 cages per group × 4 treatments) were sequenced individually. Raw sequences from all 120 samples were checked for accuracy using FastQC (https://www.bioinformatics.babraham.ac.uk/projects/fastqc/ (accessed on 28 March 2018)). Prior to analysis, we obtained a total of 6,283,142 sequences from which we kept a total of 3,141,571 reads after filtration. Reads were processed through the *dada2* pipeline (version 1.12) [73]; the quality control of reads was processed through the filterAndTrim function by using the following parameters: 270 for the read truncation length, 2 as the Phred score threshold for total read removal and a maximum expected error of 2 for forward reads and 4 for reverse reads. Filtered reads were then fed to the error rate learning, dereplication and amplicon sequence variant (ASV) inference steps using the functions learnErrors, derepFastq and dada. Chimeric sequences were removed using the removeBimeraDenovo function with the “consensus” method parameter. For information regarding the reads tracking process and the number of ASVs per sample, see Supplementary Table S1. 

#### 2.8.2. Taxonomic Assignment 

Taxonomic classification was carried out using blast matches from the NCBI 16S Microbial database [74]. As the NCBI database for 16S sequences is updated more frequently than other sources, it met our requirements for exhaustive information about lesser-known taxa, while minimizing ambiguous annotations. Matches above 98% identity were assigned the reported taxonomic identity. Sequences with no matches above the identity threshold were assigned taxonomy using a lowest common ancestor method generated from the top 50 blast matches obtained. This method is closely inspired by the LCA algorithm implemented in MEGAN [75]. 

#### 2.8.3. Alpha Diversity 

The assumption of normality was measured using a Shapiro–Wilk test [66]. Because the data distribution was not Gaussian, the Kruskal–Wallis test (KW) was used for multiple group comparison. After the KW test, a post hoc analysis (Dunn’s test) was performed to determine any significant differences between the different clothianidin concentrations tested in this study. Significance was assessed with a false discovery rate adjustment test using the Benjamini–Hochberg method [76]. Differential activity analysis performed using the DESeq2 package (v.1.30.0) [77] was used to determine statistically significant differences for ASVs with differential activity (*p* < 0.05) between the unexposed group (control) and the group exposed (experimental; 0.1, 1 and 10 ppb) to clothianidin in each honeybee gut section. 

#### 2.8.4. Network Analysis

Co-abundance networks were built using Rstudio (Version 1.3.1093) to identify significant taxon–taxon correlations in each gut section per experimental condition. Data were divided into subsets by condition (gut section + concentration). Correlation matrices were constructed using the *Hmisc* R (version 4.2-0) [78] package with *p*-values corrected with the false discovery rate (FDR) method [76]. The function rcorr() (in *Hmisc* package) was used to compute a matrix of Spearman’s rho rank correlations coefficients. It provides both the correlation coefficients and the *p*-value of the correlation for all possible pairs of columns in the ASV table. Afterwards, a false discovery rate test “Bonferroni” was applied to confirm the significance of correlation pairs. Pairs of ASVs with R Spearman coefficients ≥ 0.4 and ≤−0.4 and Corr *p*-value < 0.05 and Bonferroni *p*-value < 0.05 were considered as significant. The Spearman coefficient method is solid and comparable to the new methods of mutual information in inferring correlation [56]. Twelve microbial networks were generated from pairwise correlations between the sum of functional activity for each taxon (summarized to the genus level), and visualized using the software Cytoscape (version 3.7.2) [79]. Each node represents a bacterial genus. The size of each node is proportional to genus functional activity. The darker the node color, the more interconnected it is in the network. For network interpretation, we took in consideration (*i*) edge with significant positive or negative correlations according to Spearman’s correlation coefficient such that −1 ≤ r ≤ −0.4 (negative, red edge) and 0.4 ≤ r ≤ 1 (positive, green edge); *p*-value < 0.05 with FDR correction; and (*ii*), we only accounted for bee gut taxa that occurred in most replicated samples (*n* > 7 on 10 samples per condition). We defined low activity taxa as genera with a very low activity (<0.01%) of the total sample activity that occurred in very few samples (*n* < 3 on 10 per condition). 

To further assess significant gut bacterial disturbances induced by clothianidin, we measured the distribution of the following network topological parameters: Degree (DG), Neighborhood Connectivity (NC) and Closeness Centrality (CC) obtained with the Network Analyser function built into Cytoscape. DG is a local quantification representing the communication activity inside a network. The more the DG of a node increases, the more a node is connected locally inside the network [80,81]. NC is a quantitative score providing the average connectivity of a specific node to the overall network. The more the NC increases, the more this node will impact the global network dynamics [82,83]. CC is a qualitative measure representing the mean of the shortest path length. A high CC means that the node is central inside the network and can interact with the other nodes [84].

## 3. Results

### 3.1. Honeybee Survival Is Unexpectedly Lower When Exposed to Lesser Concentrations of Clothianidin

**Figure 1:**
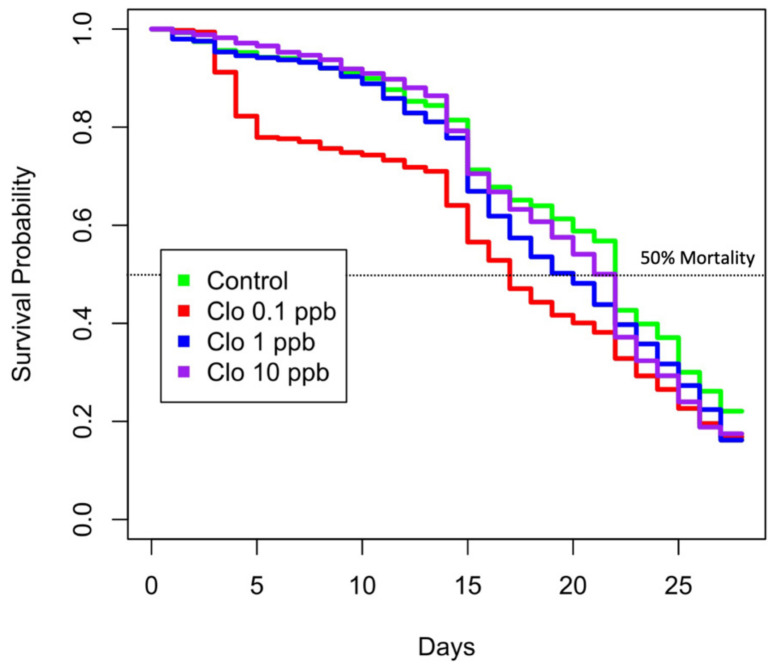
Kaplan–Meier survival curves of bees in each experimental group during the 28-day cage experiment. The y-axis represents the Kaplan–Meier estimates of the survival probability. The *x*-axis represents the experimental days. The red, blue and violet curves represent survival probability of honeybees exposed to 0.1, 1 and 10 ppb clothianidin, respectively. The green curve represents the survival rate of honeybees supplemented with 50%_w/v_ sucrose solution only.

Our results indicated a significant difference between the survival rate of the group exposed to 0.1 ppb clothianidin and the control group at the beginning of the experiment, with a trend that was shown to decrease over time (Table 1). The other exposure levels (1 and 10 ppb) did not show significant difference with the control group at the beginning of the experiment. However, a significant difference of the survival rates between these two groups increased with time (Table 1). These observations are endorsed by Kaplan–Meier survival curves in honeybees in Figure 1. Honeybee survival was inversely proportional to the clothianidin concentration: bees exposed to 0.1 ppb of clothianidin had the highest mortality relative to 1 and 10 ppb groups. Similarly, bees exposed to 1 ppb had higher mortality compared to bees exposed to 10 ppb (Figure 1). For bees exposed to 0.1 ppb, survival probability was 19.6%, 23.7% and 30.4% less than the control (unexposed group) on days 7, 14 and 21, respectively (Figure 1), and significantly different from control at all time points (Supplementary Materials Table S2). Similarly, honeybees exposed to 1 ppb showed significant differences of survival probabilities from the second week of the experiment with 7.8% and 17.1% less than the unexposed group, respectively, on days 14 and 22 (Figure 1) (Supplementary Materials Table S2). Honeybees exposed to 10 ppb showed significant differences in survival probabilities only at the beginning and at the end of the experiment (Supplementary Materials Table S2). Finally, as shown in Figure 1, honeybees exposed to 0.1 ppb reached 50% mortality at T 16 of the experimental assay. Then, bees exposed to 1 ppb reached 50% mortality at T 20. Unexposed honeybees and bees exposed to 10 ppb reached 50% mortality at T 22.

### 3.2. Feeding Rate of Honeybees Depends on Sublethal Clothianidin Concentration

**Figure 2:**
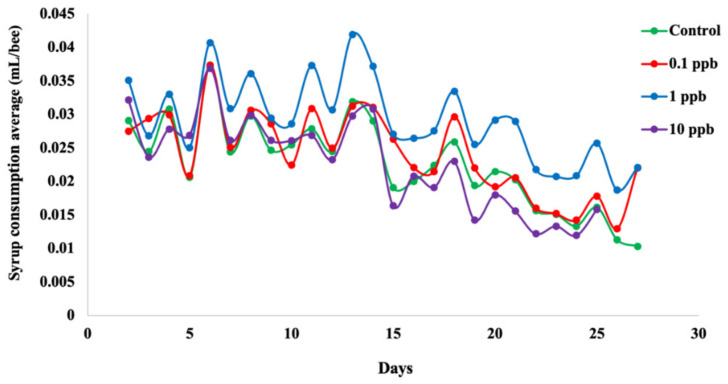
Syrup consumption in each experimental group during the 28-day cage experiment. Clothianidin effect on syrup consumption was analyzed using a non-parametric Wilcoxon test in R. The *x*-axis represents the experimental days. The red, blue and violet curves represent the syrup consumption of honeybees exposed relative to 0.1, 1 and 10 ppb clothianidin. The green curve represents the syrup consumption of honeybees supplemented with 50%_w/v_ sucrose solution only. Each dot represents the average of the syrup consumption per honeybee per group (5 cages per group, 200 worker bees per cage) measured as followed: mean of the total measured syrup consumption per cage divided by the average of the sum of living bees present at (T = t − 1) and (T = t) per cage.

Syrup consumption average per honeybee differed in all groups (Figure 2), with a significant difference between control and all exposed groups (Wilcoxon test, *p* < 0.001), suggesting a treatment effect on feeding rates (Supplementary Materials Table S3). Bees from the 1 ppb group consumed more syrup than the other experimental groups (Figure 2) and exhibited a significant difference at T 25 relative to the control (*p* < 0.05) (Supplementary Materials Table S4). For bees exposed to 0.1 ppb, we observed only a significant difference of the syrup consumption average per honeybee at T 6 relative to the control (*p* < 0.05) (Supplementary Materials Table S4). However, honeybees exposed to 10 ppb showed no significant differences in the syrup consumption average per honeybee at any time points relative to the control (Supplementary Materials Table S4). 

### 3.3. Mean Clothianidin Quantification in Experimental Honeybees

Mean clothianidin concentration accumulated (per honeybee) varied differently across all experimental groups (Supplementary Materials Table S5). At T 7, bees from the 0.1 ppb group accumulated more clothianidin than bees from the 1 and 10 ppb groups. At T 14, mean clothianidin concentration increased in the 10 ppb group in comparison to the 0.1 and 1 ppb groups. Mean clothianidin concentration in the 0.1 and 1 ppb groups decreased over time.

### 3.4. Clothianidin-Induced Changes to the Taxonomic Distribution of Intestinal Microbiota Activity

**Figure 3:**
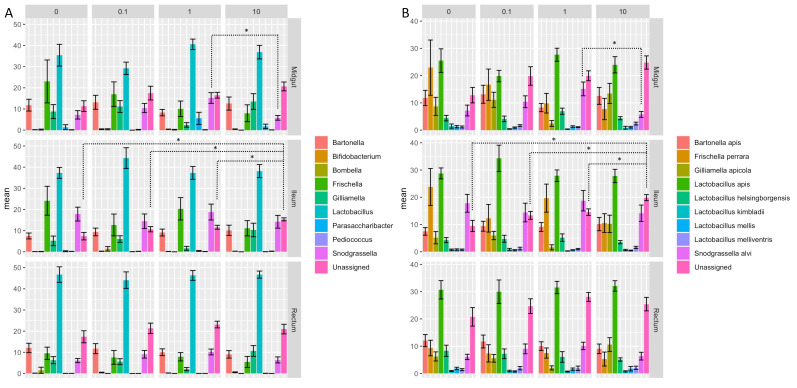
Mean of the relative activity of the 10 most dominant microbial ASVs summarized at (**A**) the genus and (**B**) species level in the honeybee gut sections (midgut, ileum and rectum) isolated from bees exposed only to sucrose syrup (0 ppb) and to three clothianidin pesticide concentrations (0.1, 1 and 10 ppb). Significant differences between the mean of the relative activity of the 10 most dominant microbial ASVs among the different experimental groups were calculated using pairwise comparisons in a Wilcoxon rank test (*p* < 0.05) corrected with the FDR method. *n* = 10 replicates per experimental condition (2 pools of 5 worker bees per cage; 5 cages per group, 50 bees per group). “*” *p* <0.05.

**Figure 4:**
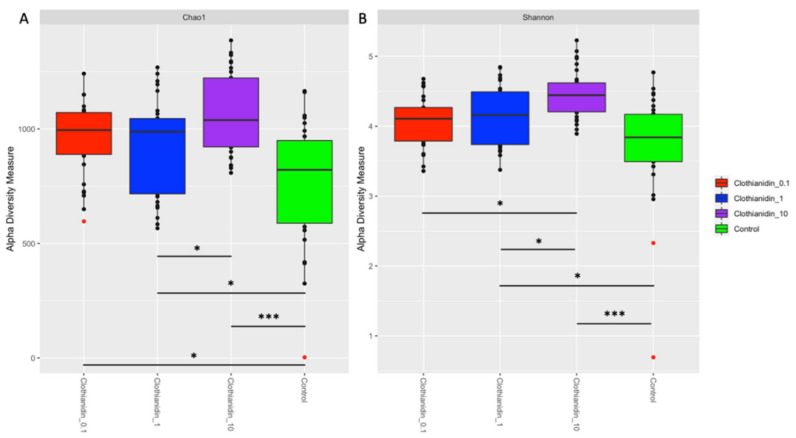
Bar plots showing the alpha diversity measures of (**A**) Chao1 estimated species richness and (**B**) Shannon diversity in all honeybee gut sections exposed to different clothianidin (0.1 ppb). We used 2 pools (2 replicates) of 5 workers per cage; 3 cages per group; 10 replicates per experimental condition. Significant differences between the alpha diversity measures of the different experimental groups were calculated using the Kruskal–Wallis test (*p* < 0.05) followed by a Dunn’s test (*p* < 0.05), the *p*-values were adjusted with the Benjamini–Hochberg correction method. “*” *p* < 0.05; “***” *p* < 0.001.

Clothianidin exposure impacted the relative contribution to microbiota activity of the ten most active ASVs (*Bartonella*, *Bifidobacterium*, *Bombella*, *Frishella*, *Gilliamella*, *Lactobacillus*, *Parasaccharibacter*, *Pediococcus*, *Snodgrassella* and unassigned ASVs) in all treatment groups versus the unexposed group (control) at the genus (Figure 3A) and species (Figure 3B) levels. Based on the relative transcriptional activity, assessment of alpha diversity in the intestinal microbial communities revealed higher richness (Figure 4A) and higher evenness (Figure 4B) regardless of gut section for honeybees exposed to clothianidin when compared to the intestinal microbiota of unexposed (control) honeybees. Our observations (Figure 4A,B) suggest a significant clothianidin dose effect on the taxonomic diversity of gut bacterial symbionts contributing to gut microbiota activity. 

### 3.5. Clothianidin Differentially Impacts the Activity of Certain Taxa at Specific Concentrations 

**Figure 5:**
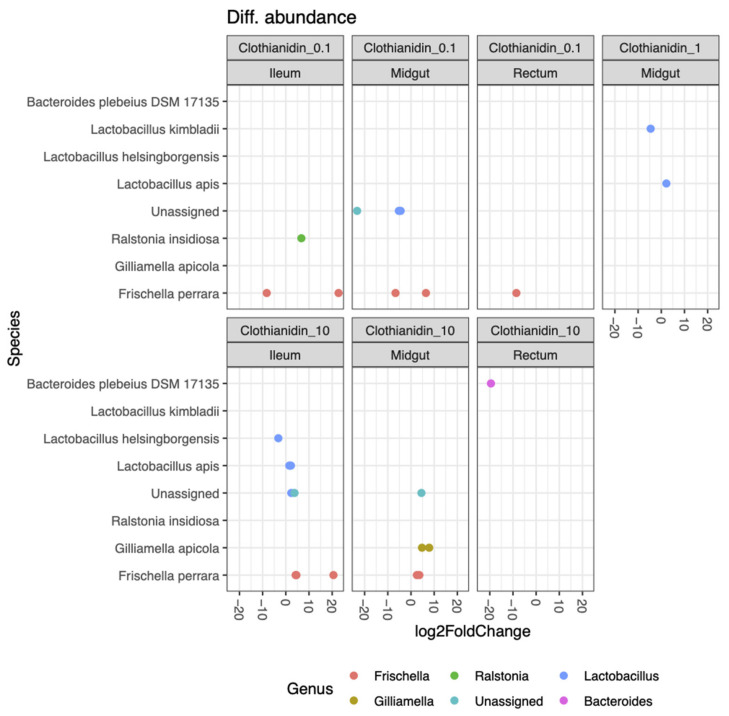
Differential activity of ASVs (total) that were significantly different (*p* < 0.05) between each experimental gut section (midgut, ileum and rectum) exposed at 0.1 ppb, 1 ppb and 10 ppb compared with the unexposed group (control) (10 replicates per experimental condition). Negative fold change scores (log2) indicate genera with decreased activity in Clothianidin-treated samples, and positive fold change scores indicate genera with increased activity. Each point represents an ASV. Only significant difference in genera activity (Adj-*p* < 0.05) is shown.

Differential activity of ASVs depends on the clothianidin concentration. The lowest clothianidin concentration (0.1 ppb) had the greatest impact on all three honeybee gut sections (Figure 5). 

The most impacted ASV in 0.1 ppb (midgut), with a significant increase and/or decrease in activity, were the two *Frischella perrara* ASVs, two *Lactobacillus spp.* and an unassigned ASV (Figure 5). The most impacted ASV in 0.1 ppb (ileum), with a significant increase and/or decrease in activity, were two *Frischella perrara* ASVs and a *Ralstonia insidiosa* ASV. At 0.1 ppb within the rectum, the only impacted ASV was *Frischella perrara*, with a significant moderate decrease in activity. At 1 ppb, *Lactobacillus apis* ASV showed a significant increase in activity; *Lactobacillus kimbladii* ASV showed a significant decrease in activity (Figure 5). 

The most impacted ASV at 10 ppb (midgut), with a significant increase and/or decrease in activity were two *Gilliamella apicola* ASVs, two *Frischella perrara* ASVs and an unassigned ASV (Figure 5). The most impacted ASV at 10 ppb (ileum), with a significant increase and/or decrease in activity were two *Frischella perrara* ASVs, a *Lactobacillus apis* ASV, a *Lactobacillus* sp. ASV, a *Lactobacillus helsinborgensis* ASV and an unassigned ASV (Figure 5). Regarding the taxonomic distribution of active ASVs contributing to the overall bacterial activity, we observed a similar number of ASVs in the three gut sections (Supplementary Materials Tables S6–S17). 

### 3.6. Clothianidin Disturbs Taxon–Taxon Interactions in the Honeybee Gut Microbiota

All the results presented below are summarized in Supplementary Materials Tables S18–S30.

**Figure 6:**
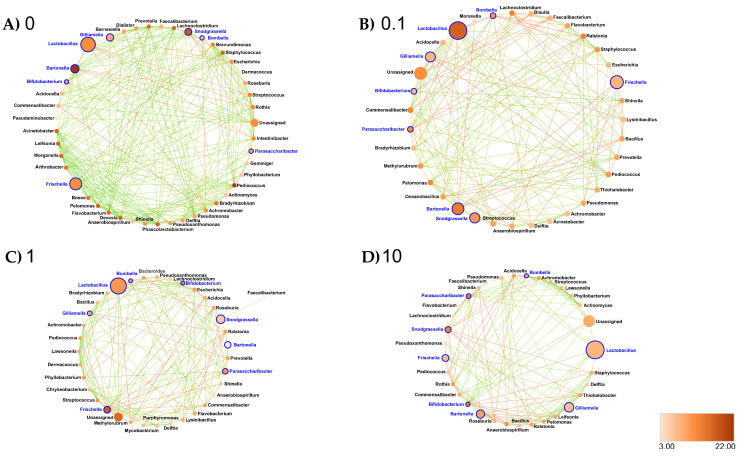
Interaction networks were generated based on pairwise correlations between the relative abundance of different bacterial genera for the midgut exposed at (**A**) 0 ppb, (**B**) 0.1 ppb, (**C**) 1 ppb and (**D**) 10 ppb. We used 10 replicates (5 workers per replicate) per experimental condition. Each node represents a bacterial genus. The size of each node is proportional to the bacterial functional activity of each genus. The darker the node, the more interconnected it is. Each edge represents significant positive or negative Spearman correlation coefficients (−1 ≤ r ≤ −0.4) (negative, red) and (0.4 ≤ r ≤ 1) (positive, green); (FDR-adjusted *p*-value < 0.05).

- Midgut: At the genus taxonomic rank, the number of interacting ASVs varied from 45 (control midgut) to 35, 36 and 33, respectively, after 0.1, 1 and 10 ppb of exposure to clothianidin (Figure 6). Significant positive correlations decreased from 96 (control midgut) to 28, 30 and 40, respectively, at 0.1, 1 and 10 ppb of exposure to clothianidin, and significant negative correlations increased from 1 (control midgut) to 5, 5 and 4, respectively, at 0.1, 1 and 10 ppb of exposure to clothianidin (Supplementary Materials Table S30). Concerning honeybee gut core members: *Lactobacillus*, *Snodgrassella* and *Gilliamella* activity was variable across concentrations. For all clothianidin treatment concentrations, *Bifidobacterium* (core members) activity increased, *Frischella* (core member) activity decreased and *Flavobacterium* (low activity taxa) decreased. Then, there was a gain in significant correlations with the following low activity ASV *Ralstonia* (0.1 and 10 ppb). Finally, there was a loss of correlations with *Devosia* (0.1 and 1 ppb) and *Leifsonia* (for all three concentrations).

In terms of network metrics, exposed midgut ASVs were significantly less connected (Low Degree, DG) relative to the control midgut network (0.1 ppb: *p* = 0.045; 1 ppb: *p* ≤ 0.001; 10 ppb: *p* = 0.002) (Supplementary Materials Figure S1 and Tables S18–S21). Regarding Closeness Centrality (CC) and Neighborhood Connectivity (NC), we observed significantly higher values for CC (*p* = 0.03 at 0.1 ppb) and significantly lower values for NC (*p* ≤ 0.001 at 0.1, 1 and 10 ppb) relative to the control midgut network (Supplementary Materials Figures S2 and S3 and Tables S18–S21). Our results support significant differences for CC between the microbial networks exposed to clothianidin (*p* = 0.012 at 1 and 10 ppb, relative to 0.1 ppb) (Supplementary Materials Figure S1). Additionally, we observed a significantly lower NC (*p* ≤ 0.001; 0.1 ppb) and a significantly higher NC (*p* = 0.025; 10 ppb) relative to 1 ppb (Supplementary Materials Figure S3 and Tables S19–S21).

**Figure 7:**
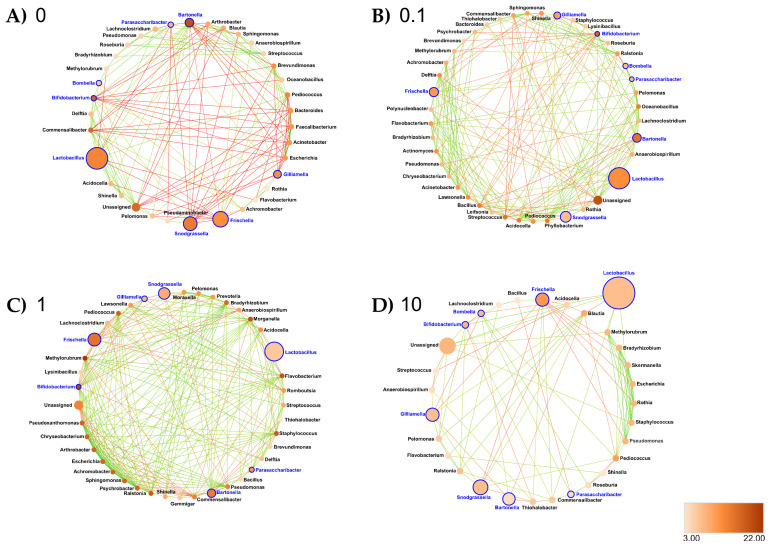
Interaction networks were generated based on pairwise correlations between the relative abundance of different bacterial genera for the ileum exposed at (**A**) 0 ppb, (**B**) 0.1 ppb, (**C**) 1 ppb and (**D**) 10 ppb. We used 10 replicates (5 workers per replicate) per experimental condition. Each node represents a bacterial genus. The size of each node is proportional to the bacterial functional activity of each genus. The darker the node, the more interconnected it is. Each edge represents significant positive or negative Spearman correlation coefficients (−1 ≤ r ≤ −0.4) (negative, red) and (0.4 ≤ r ≤ 1) (positive, green); (FDR-adjusted *p*-value < 0.05).

- Ileum: At the genus taxonomic rank, the number of interacting ASVs varied from 35 (control ileum) to 42, 40 and 30, respectively, after 0.1, 1 and 10 ppb of exposure to clothianidin (Figure 7). Significant positive correlations increased from 27 (control ileum) to 52, 73 and 29 at 0.1, 1 and 10 ppb (Figure 7, Supplementary Materials Table S30). Regarding negative correlations, significant negative correlations decreased from 6 (control ileum) to 5, 4 and 2, respectively, at 0.1, 1 and 10 ppb of exposure to clothianidin (Figure 7, Supplementary Materials Table S30). Concerning honeybee gut core members, *Lactobacillus* and *Gilliamella* activity was variable across all treatment concentrations. For all concentrations, *Bifidobacterium* (core members) activity increased and *Snodgrassella* and *Frischella* (core members) activity decreased. We observed a gain in significant correlations with low activity ASVs: *Moraxella* and *Prevotella* (1 ppb); *Lawsonella* (0.1 and 1 ppb) and *Ralstonia* (for all three concentrations). At 0.1 ppb, we observed a slight increase in *Pseudomonas* and *Flavobacterium* taxa activity.

In terms of network metrics, exposed ileum networks were significantly more connected (Higher Degree, DG) (*p* = 0.018) at 1 ppb and significantly less connected (*p* = 0.002) at 10 ppb compared to the control ileum network, respectively (Figure 7; Supplementary Materials Figure S4). Regarding Closeness Centrality (CC) and Neighborhood Connectivity (NC), there was a significantly lower CC (*p* = 0.023) and lower NC (*p* ≤ 0.001) at 1 ppb relative to the control ileum network, respectively (Supplementary Materials Figures S5 and S6 and Tables S22 and S24), and a significantly higher CC (*p* = 0.001) and higher NC (*p* ≤ 0.001) at 10 ppb, respectively, relative to the control ileum network (Supplementary Materials Figures S5 and S6 and Tables S22 and S25). Our results also support significant differences between the microbial networks exposed to clothianidin. There was a significantly lower CC (*p* = 0.001) at 1 ppb relative to 0.1 ppb (Supplementary Materials Figure S5 and Tables S23 and S24); there was a significantly higher CC (*p* ≤ 0.001) at 10 ppb relative to 1 ppb (Supplementary Materials Figure S5 and Tables S24 and S25); there was a significantly lower NC (*p* ≤ 0.001) at 1 ppb relative to 0.1 ppb (Supplementary Materials Figure S6 and Tables S23 and S24); there was a significantly higher NC (*p* ≤ 0.001) at 10 ppb relative to 0.1 ppb (Supplementary Materials Figure S6 and Tables S23 and S25); there was a significantly higher NC (*p* ≤ 0.001) at 10 ppb relative to 1 ppb (Supplementary Materials Figure S6 and Tables S24 and S25). At 0.1 ppb, *Snodgrassella* was positively correlated with *Roseburia,* a low activity ASV; *Ralstonia* was positively correlated with a low activity ASV, *Commensalibacter* (Figure 7). At 1 ppb, *Ralstonia* experienced an increase in DG, CC and NC (Supplementary Materials Table S24). In addition, *Bifidobacterium* was positively correlated with a low activity ASV, *Ralstonia,* and a probiont like *Snodgrassella*, and negatively correlated with a probiont like *Gilliamella*; *Gilliamella* was negatively correlated with *Bifidobacterium* and *Snodgrassella; Ralstonia* was positively correlated with the low activity ASVs *Pelomonas* and *Prevotella* (Figure 7). At 10 ppb, *Frischella* was negatively correlated with *Snodgrassella*; *Lactobacillus* was positively correlated with *Bifidobacterium*; *Gilliamella* was positively correlated with a low activity ASV, *Pelomonas;* and *Parasaccharibacter* was positively correlated with *Roseburia,* a low activity ASV; *Ralstonia* was positively correlated with *Pelomonas* and *Flavobacterium* (Figure 7).

**Figure 8:**
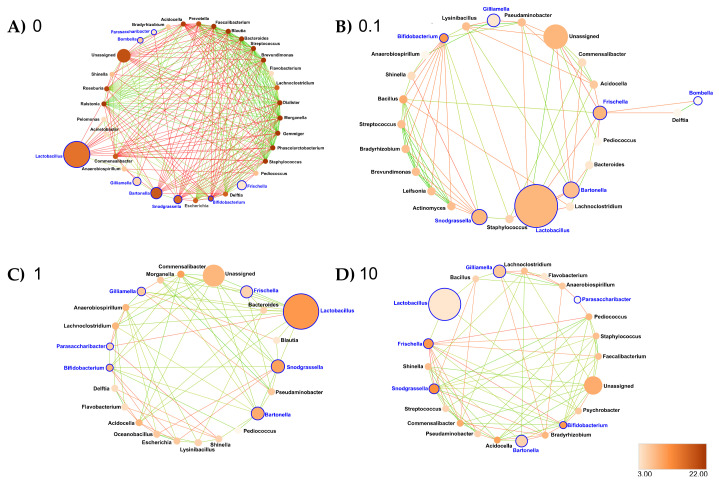
Interaction networks were generated based on pairwise correlations between the relative abundance of different bacterial genera for the rectum exposed at (**A**) 0 ppb, (**B**) 0.1 ppb, (**C**) 1 ppb and (**D**) 10 ppb. We used 10 replicates (5 workers per replicate) per experimental condition. Each node represents a bacterial genus. The size of each node is proportional to the bacterial functional activity of each genus. The darker the node, the more interconnected it is. Each edge represents significant positive or negative Spearman correlation coefficients (−1 ≤ r ≤ −0.4) (negative, red) and (0.4 ≤ r ≤ 1 ) (positive, green); (FDR-adjusted *p*-value < 0.05).

- Rectum: At the genus taxonomic rank, the number of interacting ASVs varied from 34 (control rectum) to 25, 23 and 22, respectively, after 0.1, 1 and 10 ppb of exposure to clothianidin (Figure 8). We observed a strong decrease in significant positive correlations, from 108 (control rectum) to 21, 13 and 17, respectively, at 0.1, 1 and 10 ppb of exposure to clothianidin, and a decrease in significant negative correlations from 5 (control rectum) to 1, 0 and 3, respectively, at 0.1, 1 and 10 ppb of exposure to clothianidin (Figure 8, Supplementary Materials Table S30). Concerning honeybee gut members: *Lactobacillus*, *Snodgrassella* and *Gilliamella* (core members) activity was variable across treatment concentrations. For all concentrations, *Bifidobacterium* (core member) activity increased, while *Frischella* (core member) activity decreased.

In terms of network metrics, exposed rectum networks were significantly less connected (DG) compared to the control rectum network (*p* ≤ 0.001, *p* = 0.004 and *p* = 0.001, respectively, at 0.1, 1 and 10 ppb) (Supplementary Materials Figure S7). Then, there was a significantly lower CC (*p* ≤ 0.001, *p* = 0.028 and *p* = 0.008, respectively, at 0.1, 1 and 10 ppb) and a significantly lower NC (*p* ≤ 0.001, respectively, at 0.1, 1 and 10 ppb) relative to the control rectum network (Supplementary Materials Figures S8 and S9 and Tables S26–S29). Our results support a significantly higher CC in microbial networks exposed to 1 and 10 ppb of clothianidin relative to 0.1 ppb (*p* = 0.003 and *p* = 0.008, respectively, at 1 and 10 ppb) (Supplementary Materials Figure S8 and Tables S27–S29).

## 4. Discussion

First, exposure to a gradient of three doses of clothianidin left different signatures of microbiota dysbiosis in the three gut sections of honeybees. Changes in correlations in each gut section reveal a rise in beneficial ASVs with probiotic properties, which offsets the activity increase of spikes of potential opportunistic strains. Such dysbiosis patterns were expected, given as gut sections are colonized by specific microbial communities [32]. In the midgut and the rectum, an overall decrease in ASVs activity correlation was detected in all groups exposed to clothianidin. In the ileum, two types of variations occurred: significant correlations among ASVs increased in 0.1 and 1 ppb groups and decreased in the 10 ppb group.

We expected that exposure to clothianidin would significantly impact the gut microbiota network structure by increasing positive and negative correlations between putative opportunistic strains [56,85] by a change in correlation type between core members [57] due to a direct and/or an indirect clothianidin toxicity to microbes [86] and by variations occurring in the alpha diversity readouts [56]. Despite disrupted microbial activity correlations, dominant core and non-core members were still active in all test groups (Figure 3), as reported in a previous study focusing on ASV abundance [87]. Our results indicate that exposure to clothianidin (three concentrations) had a different impact on the activity of honeybee gut-specific ASVs. The respective activities of *Lactobacillus* Firm-5 and *L.* Firm-4 clades [88,89] changed depending on the gut section and the pesticide concentration (0.1, 1 and 10 ppb). Within the honeybee rectum, which is known to be mostly dominated by *Lactobacillus* Firm-5 and *L.* Firm-4 clades [30], we detected changes in *Lactobacillus* activities at 0.1 (Supplementary Materials Tables S14 and S15), 1 ppb (Supplementary Materials Tables S14 and S16) and 10 ppb of clothianidin (Supplementary Materials Tables S14 and S17)*. Gilliamella apicola,* which is known to be abundant within the honeybee ileum gut [30], was shown to be strongly impacted at 1 ppb, with a decrease in activity of 67.2% (Supplementary Materials Tables S10 and S12). The lowest clothianidin concentration (0.1 ppb) induced a 13% decrease in *G. apicola* activity (Supplementary Materials Tables S10 and S11), while the highest concentration (10 ppb) induced a 58.75% increase in *G. apicola* activity (Supplementary Materials Tables S10 and S13). 

Interestingly, our study highlights that microbiota gut dysbiosis does not necessarily translate into a decrease in alpha diversity. The increase in alpha diversity that occurred for the three concentrations of clothianidin (Figure 4) is consistent with a previous study resulting in increased alpha diversity and a surge in negative correlations in the interacting network in yellow perch [56]. Both of these studies contrast with two previous studies in honeybee gut microbiota that found either a decrease in alpha diversity after exposure to antibiotics [90] or no difference after exposure to imidacloprid [49].

Second, the clothianidin–microbiota interaction induced a toxicity tolerance scenario. The microbial strain that first metabolizes the molecule into an intermediate molecule, or a derivate influences the syntrophic exchange network. For instance, honeybee gut microbiota exposed to two fipronil concentrations did not respond similarly: the lowest concentration (0.25 μg/kg) affected *Bifidobacterium* sp. abundance, with no significant bee mortality increase, whereas the highest concentration (1 μg/kg) did not affect *Bifidobacterium* sp. abundance, but induced a significant increase in bee mortality [8]. Daisley et al. [48] showed in gnotobiotic *Drosophila* that the pesticide chlorpyrifos was more toxic than its metabolite (chlorpyrifos oxon). In our case, the final toxicity of metabolites based on the initial clothianidin concentration could differentially impact honeybee physiology, and in turn, survival.

Interestingly, clothianidin quantification with LC-MS/MS suggests a slower degradation at 0.1 ppb (Supplementary Materials Table S5), which could result from a different clothianidin metabolization pathway, potentially translating into higher toxicity, and thus a lower survival rate. Previous studies reported clothianidin degradation by *Flavobacterium* and *Pseudomonas* sp. [44] and imidacloprid degradation by *Leifsonia* sp. [43]. Interestingly, activity of *Flavobacterium* and *Pseudomonas* increased in the ileum of 0.1 ppb group (Supplementary Materials Tables S22 and S23), where the highest mortality rate was recorded (Figure 1), and to a lesser extent in the 1 ppb group (Supplementary Materials Tables S22–S24). In a recent study on honeybee gut dysbiosis, activity of *Flavobacteriales* was increased in *N. ceranae*-infected group exposed to 0.5 µg/L fipronil [91]. Additionally, infection of *Drosophila* with a distinct *Pseudomonas* strain enhanced gut dysbiosis [92]. In a vertebrate model, an increase in abundance of *Flavobacterium* and *Pseudomonas* taxa was observed following copper exposure [93]. Further analysis on metabolites generated by *Flavobacterium* spp. and *Pseudomonas* spp. grown in media with a clothianidin concentration gradient might yield more information on their possible ability to modulate clothianidin toxicity in the honeybee. Second, the significant changes that occurred within the overall exposed gut microbial community may also depend on direct and/or indirect toxicity induced by the different pesticide concentrations and characterized by changes in the interactions among microbes [86,94,95] as illustrated by our results (Figure 6, Figure 7 and Figure 8). 

A previous study [96] showed that nutritional stress in honeybees primarily targeted ileum microbiota, translating into the highest level of dysbiosis in this gut section. They pointed out a significant increase in the intrinsic pathogen *Frischella* combined with an increase in the non-core ileum bacteria *Parasaccharibacter apium*. In concordance with our study, we highlighted an increased activity of a *Frischella* ASV in the ileum at 0.1 and 10 ppb (Figure 5), which was correlated with an increase of interactions with low activity taxa potentially opportunistic strains and/or with probiotic properties (Figure 7). Gut microorganisms have the ability to modify host immunological activity, which may have an indirect effect on other gut microorganisms and host fitness [32]. Increase of *Frischella perrara* activity in the ileum may be a direct immune host response in reaction to a microbial gut disbalance [97] in reaction to exposure to clothianidin, in our case. We also highlighted a positive correlation between *Roseburia* sp., a low activity taxon, with core (*Snodgrassella* at 0.1 ppb) and non-core (*Parasaccharibacter* at 10 ppb) gut microbes in the ileum (Figure 7). *Roseburia* sp. has shown to be involved in SCFAs production to enhance immunity in a vertebrate host [93]. It is possible that increase of *Roseburia* sp. interactions with more abundant symbionts in clothianidin-exposed ileum may be a direct immune response to pathogenic invasion. For example, an increase of the *Ralstonia* genus was shown to induce a great impact in the ileum networks at all three concentrations (0.1, 1 and 10 ppb) (Figure 7B–D) with a greater impact in the ileum (0.1 ppb) (Figure 5) where the highest mortality rate was recorded (Figure 1).

Collectively, all these changes in the microbiota structure support a dysbiosis signature in the ileum gut microbiota in our study and may suggest a clothianidin’s possible relation to the honeybee microbiota–immunity axis. In [96], the authors hypothesized that ileum dysbiosis reflected on other gut sections, inducing a systemic dysbiosis impact on the overall honeybee gut microbiota. However, evidence is lacking in our experiment to argue that different dysbiosis signatures observed in the midgut and the rectum (all three concentrations) (Figure 6 and Figure 8) are resulting from the disbalance occurring in the ileum exposed to sublethal dose of clothianidin (Figure 7). 

To summarize, clothianidin differentially impacted the activity of certain ASVs at specific concentrations, disturbed ASV–ASV interactions in the honeybee gut microbiota and properties of clothianidin degradation are determined by specific ASV. Likewise, different members may have handled the molecule first, determining excreted metabolites and thus toxicity, which may not be proportional to the initial pesticide concentration.

Third, low activity ASVs exhibit keystone species properties in honeybee gut microbiota. Our work highlights the importance of low activity ASVs, previously shown to ensure stability [81,98,99,100,101], as keystone species in the gut microbiota. For instance, there was a noticeable loss of interactions with *Leifsonia* (0.1 and 1 ppb) and *Devosia* (for all three concentrations) relative to the control midgut (Figure 6), in which these taxa are characterized by a high degree of connectivity based on the network metrics (Supplementary Materials Table S18), therefore supporting their status as keystone species in honeybee gut microbiota. A *Leifsonia* strain was isolated from honeybee brood comb and hive floor in Uruguay [102]. Interestingly, another *Leifsonia* strain was also found in agricultural soil and shown to be able to degrade imidacloprid, a neonicotinoid pesticide [51,103]. *Devosia* strains were previously isolated from insect’s environment, specifically from the floral nectar of the herb *Pulmonaria officinalis*, which is known to be pollinated by bees, in Belgium [104], from the gut microbiota of the Asian insect ladybird *Harmonia axyridis* [105], from the soil microbiome [106] and, finally, another *Devosia* strain was discovered with an obligatory plant ant, *Pseudomyrmex ferrugineus*, in Mexico [107]. 

Fourth, low activity taxa showed their implications in the honeybee gut eubiosis/dysbiosis microbiota. To understand why the lowest clothianidin concentration induced the lowest honeybee survival rate, we investigated whether a specific signature of microbiota dysbiosis could be associated with the lowest clothianidin concentration (0.1 ppb) treatment group. Loss of positive correlations with increasing neonicotinoid concentrations along intestinal sections indicates dysbiosis [108]. 

As stated above, the most extensive adverse impact, in terms of correlational network structure, was recorded at 0.1 ppb (Figure 6, Figure 7 and Figure 8). For instance (in the rectum), for *Bifidobacterium*, *Pediococcus* and *Commensalibacter* are known either as core microbiota members or as probiotics [15,109,110]. Furthermore, numerous correlations occurred with *Lawsonella,* a low activity taxa, thus supporting its role as a keystone species inside the ileum (Supplementary Materials Tables S22 and S23). In this respect, *Lawsonella* strains were also detected in the healthy gut microbiota of *Phasmotaenia lanyuhensis*, an insect [103], suggesting a potential beneficial role in honeybee gut microbiota. In the midgut, numerous correlations occurred with low activity taxa (Figure 6), translating into high NC (Supplementary Materials Table S19) for *Moraxella* spp. [111], which suggests another positive effect on overall network connectivity. *Moraxella* strains belong to the Moraxellaceae family, and were previously isolated from the intestinal giant Asian honeybee *Apis dorsata* in low abundance (0.5%) [107], the herb *Pulmonaria officinalis* floral nectar [98] and, finally, from the intestinal honeybee *Apis mellifera*, where they exhibited an antimicrobial resistance to the bacterial pathogen *Paenibacillus larvae* [111]. The other taxa identified in this study have not been well studied and were not reported in previous studies on honeybee gut microbiota.

Fifth, our study highlights a local honeybee gut microbiota reaction to exposure to clothianidin. Within all three gut sections, we observed a gain in significant correlations (positive and/or negative) for the genera *Bifidobacterium, Frischella, Gilliamella, Lactobacillus, Parasaccharibacter* and *Snodgrassella.* Each of these bee symbionts are known to be involved in either host immunity or maintenance of a homeostatic microbiota [53,97,112,113,114]. For example, *Gilliamella apicola* [115] and *Lactobacillus* [116] are responsible for short-chain fatty acid production, and their diminishing activity likely alters the host’s immunity [117]. Moreover, the functional complementarity between *Snodgrassella alvi* and *G. apicola* ensures homeostatic microbiota in the intestinal ecosystem [114]. *Frischella perrara* [97] and *Parasaccharibacter* spp. [112] were documented as important key factors in the immune system. Investigating the local effect of clothianidin gradient on the gut microbiota structure, we found a gain in correlations (positive and/or negative) among low activity taxa. Strains of these genera have been documented as pathogenic, opportunistic or potentially beneficial, with some showing probiotic properties [111]. Therefore, in this study, low activity ASVs that were not formally identified as pathogenic or beneficial for bees are deemed potential opportunistic strains.

Exposure to 0.1 ppb clothianidin (midgut) (Figure 6B) was more harmful relative to the other concentrations (Figure 6C,D), as honeybee gut *Lactobacillus* Firm-5 and *L.* Firm-4 clades were restricted, evidenced by the significant decrease in the respective activities (Supplementary Materials Tables S6–S9) and loss of connectivity (NC) in networks (Supplementary Materials Tables S18 and S19). In contrast, the activity of the distinct *Lactobacillus* ASV increased in DG and CC (Supplementary Materials Tables S18 and S19), establishing these specific ASVs as keystone species at the lowest concentration of clothianidin (0.1 ppb). Overall, the *Lactobacillus* genus is known to improve the immune system and resistance against pathogens [21,55,118,119] as well as reduce pesticide toxicity [120]. This functional profile suggests that this *Lactobacillus* ASV activity may partly ensure physiological homeostasis during dysbiosis. 

Complementarily, loss of connectivity (diminishing DG) for *Bifidobacterium* and *Pediococcus* (diminishing CC) (Supplementary Materials Tables S18 and S19) supports an adverse impact of clothianidin on these genera known for their probiotic properties [15,109,110,121,122]. However, an increase in NC for *Bifidobacterium* may suggest a more local cooperation despite an overall loss of connectivity. Similarly, an increase in *Frischella perrara* ASV activity (Figure 5) at 0.1 ppb (midgut) supports honeybee immune system activation [97]. Taken together, the correlation patterns and network metrics may suggest a pathogenic shift compensated by mutualistic correlation (e.g., *Lactobacillus*, *Bifidobacterium* and *Pediococcus*) following exposure to clothianidin. This competition shift suggests a dysbiosis rewiring pattern [57].

Previous studies highlighted fluctuations of honeybee microbiota facing stress. Applications of coumaphos, tau-fluvalinate [123] and tetracycline [90] were shown to increase *Giamella apicola* abundance. Different experimental approaches may induce microbial composition variability [53], as observed in our work with *Giamella apicola, Snodgrassella alvi* and *Frischella perrara*. We observed a decrease and/or an increase in *Frischella perrara* activity, while exposure to other pesticides was variable, as it either induced (nitenpyram) [20] or failed to induce (imidacloprid) an increase in *F. perrara* abundance [49]. *F. perrara* is known to play a key role in honeybee immunity by limiting microbial resistance [97]; therefore, *F. perrara* disbalance may affect honeybee gut immunity, leading to microbiota dysbiosis. Finally, the increasing activity of *Bifidobacterium* activity confirms results of previous studies that tested honeybee exposure to nitenpyram and thiacloprid [19,20,21].

Additionally, *Snodgrassella alvi* is involved for the upregulation of the gene expression related to antimicrobial peptides [124]. Destabilization of the honeybee gut biofilm pioneer, *S. alvi*, may create an overall imbalance in gut microbiota. A decrease in *S. alvi* activity in the ileum following exposure to pesticide is consistent with findings of two previous studies [125,126]. In our experiment, it is likely that decreasing *S. alvi* activity played a role in adversely impacting the immune response of *Apis mellifera* when facing an increase in potential opportunistic strains such as *Roseburia* and *Pelomonas*. Our results provide additional evidence on the level of bacterial activity, showing that honeybees exposed to neonicotinoids are more sensitive to microbial gut pathogens [49,127,128]. 

More specifically, the increase of potential opportunistic ASVs activity was variable along the gut section and gradient of exposure. *Ralstonia* genus had a greater impact on the ileum (0.1, 1 and 10 ppb) networks (Figure 7B–D). For instance, high CC and NC for *Ralstonia* revealed its extensive connectivity within the overall ileum network (Supplementary Materials Tables S18–S20 and S22–S25), suggesting this strain exerts an important negative impact on microbiota wiring following exposure to clothianidin. Overall, clothianidin induced negative correlations between core (e.g., *Giamella apicola, Snodgrassella alvi* and *Frischella perrara*) and non-core members within each gut section. Given that these core symbionts are either involved in host immunity and/or microbiota equilibrium [53,97,112,113,114], our results provide a better understanding of the dysbiosis induced by exposure to clothianidin and its impact on the microbiota–immunity axis [21,22,23]. 

Sixth, this study highlights how environmental factors adversely shape individual honeybee health. The link established here between low exposure to clothianidin, high mortality and overall honeybee health differs somewhat from previous studies. Alkassab and colleagues [4] reported on laboratory and field experiments in which honeybees were exposed to clothianidin concentrations ranging between 1 and 200 μg/L, showing that the 200 μg/L exposure in field induced the highest honeybee mortality rate. Other completed experiments with honeybees exposed to sublethal clothianidin concentrations (0.5–2 μg/L) [129] (0.5–0.97 ppb) [130] did not observe significant differences between the mortality rate of honeybees exposed to clothianidin versus healthy honeybee colonies. These differences in findings may be due to the variability of honeybee colonies with seasons, where winter honeybees are less susceptible to mortality compared to summer bees [131]. Both studies [4,129] used winter honeybees, whereas Rolke and colleagues [130] used spring/summer bees. Winter honeybees seemed more resistant to clothianidin sublethal doses than summer bees. In addition, these three former studies [4,129,130] were performed under field conditions, while our work was performed under laboratory conditions. Variables present under field conditions differ from those in the laboratory, where honeybees are more susceptible to exposure to pesticides [4]. Osterman and colleagues [132] did not observe a negative impact on the survival of bees exposed to sublethal doses of clothianidin. In the second year, they observed a positive impact, with increased brood production and improved honeybee fitness. These results may be due to “hormesis”, a beneficial response to low exposure [133]. Other researchers [134] also studied the individual immunocompetence of honeybees exposed to clothianidin in which the antimicrobial activity of hemolymph was reduced in all clothianidin concentrations (10–200 ppb). Together, these studies showed that the impact of sublethal clothianidin concentrations on individual honeybees depends on many environmental factors. 

Finally, this study showed how honeybees are significantly more attracted by syrup supplied with the medium sublethal clothianidin concentration. In a previous study, honeybees were found to be more attracted to contaminated than non-contaminated syrup [135]. A dose-dependent attraction was observed for the neonicotinoid nitenpyram, where food consumption was negatively correlated with pesticide concentration: normal with 3–30 μL/L and low with 300 μL/L [20]. In our study, honeybees exposed to 1 ppb clothianidin consumed significantly more syrup, translating into the lowest pesticide bioaccumulation in the 1 ppb group, relative to the other groups (Figure 2, Supplementary Materials Table S5). This low clothianidin bioaccumulation may suggest that the honeybee gut microbiota may have used clothianidin as a nutrient [136,137], translating into an intermediate effect regarding survival, between 0.1 and 10 ppb groups (Figure 1, Supplementary Materials Table S2). Previous studies highlighted how nutritional stress shapes the gut microbiota composition [136,137], resulting in long-term negative impacts on honeybee health [137]. Further analyses are needed to further understand the link between sublethal exposure to clothianidin, syrup consumption, dysbiosis gut microbiota and the high honeybee mortality documented in our work.

## 5. Conclusions

This work highlights the interplay between gut microbiota activity, food intake and exposure to pesticides. Our work provides unprecedented insights regarding the impact of clothianidin gradient on the activity interactions between core members (i.e., probionts) and non-core members, including potential opportunistic strains and the potential link of clothianidin pesticide with the microbiota–immunity axis. Overall, our results suggest that extent of gut microbiota dysbiosis depends on both xenobiotic exposure level and gut section. Finally, activity interaction networks appear to be a valuable tool to measure the impact of exposure to pesticide on microbiome community structure.

## Figures and Tables

**Table 1:** Clothianidin effect explained by a Cox model non-proportional risk analysis comparisons between the 0.1, 1 and 10 ppb relative to the unexposed experimental group.

	Coef	Exp(Coef)	Se(Coef)	Robust Se	z	*p*
0.1 ppb	1.64479	5.17992	0.22153	0.57321	2.869	0.00411 **
1 ppb	0.16670	1.18140	0.24819	0.78791	0.212	0.83244
10 ppb	−0.67473	0.50929	0.28351	0.41147	−1.640	0.10105
Logtime 0.1 ppb	−0.49352	0.61048	0.08382	0.21253	−2.322	0.02023 *
Logtime 1 ppb	0.01193	1.01200	0.09169	0.28505	0.042	0.96661
Logtime 10 ppb	0.30299	1.35390	0.10303	0.13766	2.201	0.02774 *

Likelihood ratio test = 168.2 on 6 df, *p* = < 2.2 × 10^−16^; *n* = 4262, number of events = 2461. “*” *p* < 0.05; “**”*p* < 0.01.

## Data Availability

The raw sequence reads analyzed in the current study are available in the NCBI BioProject ID repository under the number PRJNA678327.

## Supplementary Materials

The following are available online at https://www.mdpi.com/article/10.3390/microorganisms9112283/s1, Table S1: Information regarding the reads tracking process using *dada2* and the number of ASVs per sample, Table S2: Cox proportional hazards model comparisons between the survival of honeybees exposed vs. unexposed to clothianidin for 28 days, Table S3: Syrup consumption average levels per honeybee between the experimental groups compared to the control group overtime, Table S4: Syrup consumption average levels per honeybee between the experimental groups compared to the control group at specific time points, Table S5: Mean quantification of the clothianidin levels (ng/mL per bee) with the standard deviation (SD) in the honeybee gut, Table S6: Total taxonomic distribution of active ASVs in the midgut exposed at 0 ppb, Table S7: Total taxonomic distribution of active ASVs in the midgut exposed at 0.1 ppb, Table S8: Total taxonomic distribution of active ASVs in the midgut exposed at 1 ppb, Table S9: Total taxonomic distribution of active ASVs in the midgut exposed at 10 ppb, Table S10: Total taxonomic distribution of active ASVs in the ileum exposed at 0 ppb, Table S11: Total taxonomic distribution of active ASVs in the ileum exposed at 0.1 ppb, Table S12: Total taxonomic distribution of active ASVs in the ileum exposed at 1 ppb, Table S13: Total taxonomic distribution of active ASVs in the ileum exposed at 10 ppb, Table S14: Total taxonomic distribution of active ASVs in the rectum exposed at 0 ppb, Table S15: Total taxonomic distribution of active ASVs in the rectum exposed at 0.1 ppb, Table S16: Total taxonomic distribution of active ASVs in the rectum exposed at 1 ppb, Table S17: Total taxonomic distribution of active ASVs in the rectum exposed at 10 ppb, Table S18: Network metrics for gut microbiome taxa in the midgut exposed to 0 ppb clothianidin, Table S19: Network metrics for gut microbiome taxa in the midgut exposed to 0.1 ppb clothianidin, Table S20: Network metrics for gut microbiome taxa in the midgut exposed to 1 ppb clothianidin, Table S21: Network metrics for gut microbiome taxa in the midgut exposed to 10 ppb clothianidin, Table S22: Network metrics for gut microbiome taxa in the ileum exposed to 0 ppb clothianidin, Table S23: Network metrics for gut microbiome taxa in the ileum exposed to 0.1 ppb clothianidin, Table S24: Network metrics for gut microbiome taxa in the ileum exposed to 1 ppb clothianidin, Table S25: Network metrics for gut microbiome taxa in the ileum exposed to 10 ppb clothianidin, Table S26: Network metrics for gut microbiome taxa in the rectum exposed to 0 ppb clothianidin, Table S27: Network metrics for gut microbiome taxa in the rectum exposed to 0.1 ppb clothianidin, Table S28: Network metrics for gut microbiome taxa in the rectum exposed to 1 ppb clothianidin, Table S29: Network metrics for gut microbiome taxa in the rectum exposed to 10 ppb clothianidin, Table S30: Number of positive (+) and/or negative (-) interactions at the genus level in the microbial network depending on each honeybee gut section (midgut, ileum and rectum) and clothianidin concentrations (Ctrl (0 ppb), 0.1, 1 and 10 ppb), Figure S1: Violin plot of Degree from taxa in midgut microbial networks, Figure S2: Violin plot of Closeness Centrality from taxa in midgut microbial networks, Figure S3: Violin plot of Neighborhood Connectivity from taxa in midgut microbial networks, Figure S4: Violin plot of Degree from taxa in ileum microbial networks, Figure S5: Violin plot of Closeness Centrality from taxa in ileum microbial networks, Figure S6: Violin plot of Neighborhood Connectivity from taxa in ileum microbial networks, Figure S7: Violin plot of Degree from taxa in rectum microbial networks, Figure S8: Violin plot of Closeness Centrality from taxa in rectum microbial networks, Figure S9: Violin plot of Neighborhood Connectivity from taxa in rectum microbial networks.
