# Supplementary material for: Dietary Contamination with a Neonicotinoid (Clothianidin) Gradient Triggers Specific Dysbiosis Signatures of Microbiota Activity along the Honeybee (Apis mellifera) Digestive Tract
Source: Microorganisms. 2021 Nov 2;9(11):2283. doi: 10.3390/microorganisms9112283 (PMC8619528; doi:10.3390/microorganisms9112283)

Dietary contamination with neonicotinoid (Clothianidin) gradient triggers specific dysbiosis signatures of microbiota activity along the honeybee (*Apis mellifera*) digestive tract

Supplementary information file

Sarah El Khoury [1,2], Jeff Gauthier [1,2], Sidki Bouslama [1,2], Bachar Cheaib [3], Pierre Giovenazzo [1], Nicolas Derome [1,2,*].

1 Departement of Biology, Laval University, Québec, QC G1V 0A6, Canada
2 Institut de Biologie Intégrative et des Systèmes (IBIS), Laval University, Québec, QC G1V 0A6, Canada
3 Institute of Biodiversity, Animal Health & Comparative Medicine, University of Glasgow G12 8QQ, United Kingdom

Contents

Table S1 : Information regarding the reads tracking process using *dada2* and the number of ASVs per sample 4

Table S2: Cox Proportional Hazards model comparisons between the survival of honeybees exposed vs. unexposed to clothianidin for 28 days 9

Table S3: Syrup consumption average levels per honeybee between the experimental groups compared to the control group overtime 9

Table S4: Syrup consumption average levels per honeybee between the experimental groups compared to the control group at specific time points 10

Table S5: Mean quantification of the clothianidin levels (ng/mL per bee) with the standard deviation (S.D.) in the honeybee gut 10

Table S6 : Total taxonomic distribution of active ASVs in midgut exposed at 0 ppb 11

Table S7: Total taxonomic distribution of active ASVs in midgut exposed at 0.1 ppb 14

Table S8: Total taxonomic distribution of active ASVs in midgut exposed at 1 ppb 16

Table S9: Total taxonomic distribution of active ASVs in midgut exposed at 10 ppb 18

Table S10 : Total taxonomic distribution of active ASVs in ileum exposed at 0 ppb 20

Table S11 : Total taxonomic distribution of active ASVs in ileum exposed at 0.1 ppb 22

Table S12 : Total taxonomic distribution of active ASVs in ileum exposed at 1 ppb 25

Table S13 : Total taxonomic distribution of active ASVs in ileum exposed at 10 ppb 27

Table S14 : Total taxonomic distribution of active ASVs in rectum exposed at 0 ppb 29

Table S15 : Total taxonomic distribution of active ASVs in rectum exposed at 0.1 ppb 31

Table S16 : Total taxonomic distribution of active ASVs in rectum exposed at 1 ppb 33

Table S17 : Total taxonomic distribution of active ASVs in rectum exposed at 10 ppb 35

Table S18 : Network metrics for gut microbiome taxa in midgut exposed to 0 ppb clothianidin 37

Table S19 : Network metrics for gut microbiome taxa in midgut exposed to 0.1 ppb clothianidin 39

Table S20 : Network metrics for gut microbiome taxa in midgut exposed to 1 ppb clothianidin 40

Table S21 : Network metrics for gut microbiome taxa in midgut exposed to 10 ppb clothianidin 42

Table S22 : Network metrics for gut microbiome taxa in ileum exposed to 0 ppb clothianidin 44

Table S23 : Network metrics for gut microbiome taxa in ileum exposed to 0.1 ppb clothianidin 46

Table S24 : Network metrics for gut microbiome taxa in ileum exposed to 1 ppb clothianidin 50

Table S25 : Network metrics for gut microbiome taxa in ileum exposed to 10 ppb clothianidin 52

Table S26 : Network metrics for gut microbiome taxa in rectum exposed to 0 ppb clothianidin 54

Table S27 : Network metrics for gut microbiome taxa in rectum exposed to 0.1 ppb clothianidin 57

Table S28 : Network metrics for gut microbiome taxa in rectum exposed to 1 ppb clothianidin 58

Table S29 : Network metrics for gut microbiome taxa in rectum exposed to 10 ppb clothianidin 60

Table S30 : Number of positive (+) and/or negative (-) interactions at the genus level in the microbial network depending on each honeybee gut section (midgut, ileum and rectum) and clothianidin concentrations (Ctrl (0 ppb), 0.1, 1 and 10 ppb) 62

Figure S1 : Violin plot of Degree from taxa in midgut microbial networks 63

Figure S2 : Violin plot of Closeness Centrality from taxa in midgut microbial networks 63

Figure S3 : Violin plot of Neighborhood Connectivity from taxa in midgut microbial networks 65

Figure S4 : Violin plot of Degree from taxa in ileum microbial networks 66

Figure S5 : Violin plot of Closeness Centrality from taxa in ileum microbial networks 66

Figure S6 : Violin plot of Neighborhood Connectivity from taxa in ileum microbial networks 68

Figure S7 : Violin plot of Degree from taxa in rectum microbial networks 69

Figure S8 : Violin plot of Closeness Centrality from taxa in rectum microbial networks 70

Figure S9 : Violin plot of Neighborhood Connectivity from taxa in rectum microbial networks 71

Table S1 : Information regarding the reads tracking process using *dada2* and the number of ASVs per sample

Sample Name	Experimental group	Input reads	Filtration step	Dereplication step	Chimera removal	Number of ASVs
1-1A	Control	33613	21532	21464	18325	615
1-1V	Control	25563	15537	15486	13298	497
1-1X	Control	26019	14838	14815	13229	326
1-2A	Control	19322	6228	6108	5674	270
1-2V	Control	41578	20508	20459	17929	197
1-2X	Control	18821	11514	11480	10507	252
2-1A	Control	21870	13824	13764	11045	581
2-1V	Control	29357	17716	17631	14891	623
2-1X	Control	24504	11738	11708	9616	399
2-2A	Control	25328	16046	15951	14558	575
2-2V	Control	26700	11595	11547	9540	578
2-2X	Control	22684	11957	11916	10189	702
3-1A	Control	27932	14444	14409	11895	480
3-1V	Control	27825	18792	18753	16161	402
3-1X	Control	25342	16878	16811	13388	726
3-2A	Control	23429	15223	15134	10520	808
3-2V	Control	20283	6707	6662	5136	527
3-2X	Control	26392	14378	14300	10965	832
4-1A	Control	22348	14183	14111	10820	849
4-1V	Control	15	5	2	2	2
4-1X	Control	24007	12648	12604	11141	499
4-2A	Control	23432	14679	14625	11928	753
4-2V	Control	21944	6905	6866	5312	560
4-2X	Control	28505	9743	9702	8292	559

5-1A	Control	25893	14671	14621	11619	746
5-1V	Control	35263	10951	10909	9718	383
5-1X	Control	45860	18083	18025	16607	400
5-2A	Control	22718	14215	14140	10731	919
5-2V	Control	28764	13177	13127	10221	401
5-2X	Control	20123	10143	10077	8106	486
6-1A	Clothianidin_0.1	27584	18413	18320	15116	796
6-1V	Clothianidin_0.1	37584	14145	14084	11401	781
6-1X	Clothianidin_0.1	39917	16315	16238	13145	718
6-2A	Clothianidin_0.1	16233	7996	7941	7052	436
6-2V	Clothianidin_0.1	32763	14725	14588	11507	767
6-2X	Clothianidin_0.1	26553	12548	12436	10746	574
7-1A	Clothianidin_0.1	23238	13248	13187	10411	765
7-1V	Clothianidin_0.1	10581	6855	6832	5529	483
7-1X	Clothianidin_0.1	20639	11033	10957	7994	775
7-2A	Clothianidin_0.1	24471	13888	13845	10415	533
7-2V	Clothianidin_0.1	26217	17021	16944	13935	704
7-2X	Clothianidin_0.1	39759	18530	18394	13410	929
8-1A	Clothianidin_0.1	29541	19188	19092	16497	531
8-1V	Clothianidin_0.1	22913	14000	13920	11699	665

8-1X	Clothianidin_0.1	22604	5054	5038	4377	352
8-2A	Clothianidin_0.1	28043	12532	12441	9078	780
8-2V	Clothianidin_0.1	22618	8179	8128	5578	648
8-2X	Clothianidin_0.1	26242	11590	11539	10032	434
9-1A	Clothianidin_0.1	30002	19123	19045	15665	616
9-1V	Clothianidin_0.1	31172	12871	12760	10189	830
9-1X	Clothianidin_0.1	24606	13738	13640	11342	805
9-2A	Clothianidin_0.1	22372	14186	14081	10216	853
9-2V	Clothianidin_0.1	24507	11185	11131	9281	693
9-2X	Clothianidin_0.1	27768	16513	16422	12872	827
10-1A	Clothianidin_0.1	20909	13211	13122	9341	816
10-1V	Clothianidin_0.1	34691	14555	14502	11965	539
10-1X	Clothianidin_0.1	34273	11859	11733	10087	593
10-2A	Clothianidin_0.1	21256	12227	12148	9216	640
10-2V	Clothianidin_0.1	20466	8383	8342	5872	675
10-2X	Clothianidin_0.1	28865	13911	13856	11737	752
11-1A	Clothianidin_1	41975	23960	23802	17786	1093
11-1V	Clothianidin_1	24817	9966	9908	7525	786
11-1X	Clothianidin_1	30467	14297	14210	11164	754

11-2A	Clothianidin_1	32112	18671	18540	13215	991
11-2V	Clothianidin_1	38629	20572	20452	15045	1043
11-2X	Clothianidin_1	34542	17916	17829	14421	863
12-1A	Clothianidin_1	21659	13446	13354	8636	881
12-1V	Clothianidin_1	38808	12745	12671	10170	676
12-1X	Clothianidin_1	33594	16764	16590	12547	811
12-2A	Clothianidin_1	18249	11080	11026	8559	785
12-2V	Clothianidin_1	23170	11395	11337	7446	714
12-2X	Clothianidin_1	25247	11731	11664	10085	500
13-1A	Clothianidin_1	15651	9447	9393	6961	799
13-1V	Clothianidin_1	23720	10964	10921	8437	681
13-1X	Clothianidin_1	15282	8049	8007	6673	494
13-2A	Clothianidin_1	17717	12175	12103	9244	704
13-2V	Clothianidin_1	18047	10469	10424	8151	635
13-2X	Clothianidin_1	17316	9906	9859	8609	528
14-1A	Clothianidin_1	28221	17378	17288	13924	575
14-1V	Clothianidin_1	21136	10562	10514	8348	455
14-1X	Clothianidin_1	21994	9321	9256	7589	418
14-2A	Clothianidin_1	15190	9606	9538	7173	493
14-2V	Clothianidin_1	24423	13912	13814	11097	504
14-2X	Clothianidin_1	17460	8017	7973	6846	354
15-1A	Clothianidin_1	10945	6539	6518	5485	395
15-1V	Clothianidin_1	21205	10765	10695	7798	587
15-1X	Clothianidin_1	21505	10931	10866	8714	518
15-2A	Clothianidin_1	28260	17115	16975	12212	902
15-2V	Clothianidin_1	22502	12827	12774	10780	515
15-2X	Clothianidin_1	27472	15506	15435	12643	690

16-1A	Clothianidin_10	16004	10573	10490	7204	719
16-1V	Clothianidin_10	19102	10672	10596	8107	632
16-1X	Clothianidin_10	18991	11496	11439	9227	645
16-2A	Clothianidin_10	17387	11090	11023	8658	604
16-2V	Clothianidin_10	21864	10631	10568	8483	589
16-2X	Clothianidin_10	26506	11667	11595	9095	684
17-1A	Clothianidin_10	33351	18797	18697	13507	907
17-1V	Clothianidin_10	38559	22461	22316	16346	1071
17-1X	Clothianidin_10	26032	11471	11380	8108	875
17-2A	Clothianidin_10	13990	8648	8596	6033	761
17-2V	Clothianidin_10	35580	15248	15128	10299	776
17-2X	Clothianidin_10	95943	26069	25890	18959	1000
18-1A	Clothianidin_10	27329	17448	17342	14320	658
18-1V	Clothianidin_10	7857	4670	4638	3276	601
18-1X	Clothianidin_10	25536	14914	14837	10498	983
18-2A	Clothianidin_10	21676	10783	10738	8449	665
18-2V	Clothianidin_10	27071	14799	14661	9257	1089
18-2X	Clothianidin_10	33271	13416	13335	10560	743
19-1A	Clothianidin_10	31118	14356	14254	9577	1049
19-1V	Clothianidin_10	23864	10494	10421	7599	954
19-1X	Clothianidin_10	27201	12648	12542	8529	702
19-2A	Clothianidin_10	11745	6436	6411	5284	542
19-2V	Clothianidin_10	26463	16568	16490	12650	778
19-2X	Clothianidin_10	26445	15723	15644	12266	965
20-1A	Clothianidin_10	18961	11581	11534	8952	558
20-1V	Clothianidin_10	32368	12709	12604	9885	783
20-1X	Clothianidin_10	49720	27283	27047	19112	1188

20-2A	Clothianidin_10	26318	14951	14873	10688	819
20-2V	Clothianidin_10	43259	19891	19761	13844	958
20-2X	Clothianidin_10	22924	12722	12638	7639	973

Table S2: Cox Proportional Hazards model comparisons between the survival of honeybees exposed *vs.* unexposed to clothianidin for 28 days

Days	6	7	14	21	22	25	26	27	28
Ctrl *vs:*									
[0.1 ppb]	< 0.05	< 0.001	< 0.001	< 0.001	< 0.001	< 0.001	< 0.001	< 0.001	< 0.001
[1 ppb]	-	-	< 0.05	-	< 0.001	< 0.05	< 0.05	-	0.001
[10 ppb]	< 0.001	-	-	-	-	-	-	< 0.05	< 0.05

Significant
P-values < 0.05 (orange);
P-values = 0.001 (green);
P-values < 0.001 (yellow).

Table S3: Syrup consumption average levels per honeybee between the experimental groups compared to the control group overtime

Treatment	[0.1 ppb]	[1 ppb]	[10 ppb]
Control	0.001	< 0.001	< 0.001

Wilcoxon rank sum test

Significant p-values < 0.05

Table S4: Syrup consumption average levels per honeybee between the experimental groups compared to the control group at specific time points

**Days**	**6**	**8**	**11**	**13**	**15**	**25**	
**Ctrl *vs:***							
**[0.1 ppb]**	< 0.05	-	-	-		-	-
**[1 ppb]**	-				-	< 0.05	
**[10 ppb]**	-	-	-	-	-	-	-

Kruskal-Wallis test

Significant p-values < 0.05

Table S5: Mean quantification of the clothianidin levels (ng/mL per bee) with the standard deviation (S.D.) in the honeybee gut

Concentration	Mean (S.D.) Day 7	R.T.	Mean (S.D.) Day 14	R.T.	Mean (S.D.) Day 21	R.T.	Mean (S.D.) Day 28	R.T.
Control	-	-	-	-	-	-	-	-
[0.1 ppb]	0.013 (0.0046)	6.26	0.009 (0.0023)	6.24	0.008 (0.0007)	6.12	0.006 (0.0039)	6.20
[1 ppb]	0.004 (0.0009)	6.19	0.007 (0.0017)	6.22	0.005 (0.0047)	6.25	0.005 (0.0038)	6.09
[10 ppb]	0.009 (0.0007)	6.20	0.021 (0.0033)	6.19	0.011 (0.0061)	6.17	0.015 (0.0063)	6.09

R.T. (Retention Time)

Table S6 : Total taxonomic distribution of active ASVs in midgut exposed at 0 ppb

ASV Species	ASV activity
[Clostridium] saccharolyticum	1
Achromobacter insolitus	23
Actinomyces johnsonii	6
Arthrobacter russicus	10
Bacteroides caccae	1
Bacteroides plebeius DSM 17135	4
Bartonella apis	6004
Bifidobacterium asteroides	31
Bifidobacterium callitrichos DSM 23973	1
Bifidobacterium coryneforme	44
Bombella apis	44
Bosea vaviloviae	7
Bradyrhizobium oligotrophicum S58	1
Bradyrhizobium vignae	27
Brevundimonas albigilva	56
Brevundimonas lenta	12
Chryseobacterium hispanicum	1
Delftia acidovorans	102
Dermacoccus profundi	4
Dialister invisus	6
Escherichia fergusonii ATCC 35469	1

Faecalibacterium prausnitzii	1
Frischella perrara	25666
Gilliamella apicola	4405
Gilliamella bombicola	1
Gilliamella intestini	17
Intestinibacter bartlettii	82
Lachnoclostridium pacaense	18
Lactobacillus apis	18339
Lactobacillus helsingborgensis	3718
Lactobacillus kimbladii	1843
Lactobacillus kullabergensis	133
Lactobacillus kunkeei	8
Lactobacillus mellifer	111
Lactobacillus mellis	1077
Lactobacillus melliventris	635
Leifsonia shinshuensis	1
Lysinibacillus parviboronicapiens	8
Moraxella osloensis	36
Morganella morganii subsp. sibonii	6
Oceanobacillus chironomi	4
Parasaccharibacter apium	270
Pediococcus stilesii	180
Pelomonas saccharophila	37
Pseudaminobacter manganicus	155
Pseudomonas canadensis	50
Pseudomonas cerasi	74

Pseudomonas songnenensis	29
Pseudoxanthomonas jiangsuensis	27
Psychrobacter cryohalolentis K5	82
Ralstonia pickettii	165
Romboutsia timonensis	519
Roseburia faecis	770
Rothia dentocariosa ATCC 17931	162
Shinella fusca	2138
Skermanella aerolata	848
Snodgrassella alvi wkB2	10265
Sphingomonas echinoides	3628
Staphylococcus epidermidis	9005
Streptococcus oralis subsp. dentisani	6983
Streptococcus salivarius	7593
Unassigned	10788

Table S7: Total taxonomic distribution of active ASVs in midgut exposed at 0.1 ppb

ASV Species	ASV activity
[Clostridium] saccharolyticum	1
Bacillus licheniformis	1
Bacillus wiedmannii	2
Bacteroides caccae	2
Bacteroides plebeius DSM 17135	1
Bartonella apis	6052
Bifidobacterium asteroides	12
Bifidobacterium callitrichos DSM 23973	1
Bifidobacterium coryneforme	23
Bombella apis	236
Bradyrhizobium oligotrophicum S58	4
Bradyrhizobium vignae	4
Dermacoccus profundi	10
Dialister invisus	4
Frischella perrara	9582
Gemmiger formicilis	0
Gilliamella apicola	2772
Intestinibacter bartlettii	2
Lachnoclostridium pacaense	20
Lactobacillus apis	3492
Lactobacillus helsingborgensis	480
Lactobacillus kimbladii	89
Lactobacillus kullabergensis	19

Lactobacillus kunkeei	1
Lactobacillus mellifer	42
Lactobacillus mellis	185
Lactobacillus melliventris	487
Lysinibacillus parviboronicapiens	10
Methylorubrum populi BJ001	1
Oceanobacillus chironomi	28
Parasaccharibacter apium	1
Pediococcus stilesii	1
Pelomonas saccharophila	71
Phascolarctobacterium faecium	114
Pseudaminobacter manganicus	9
Pseudomonas canadensis	254
Pseudomonas cerasi	114
Pseudomonas songnenensis	146
Pseudoxanthomonas jiangsuensis	17
Psychrobacter cryohalolentis K5	440
Ralstonia insidiosa	232
Ralstonia pickettii	436
Romboutsia timonensis	994
Roseburia faecis	1577
Rothia dentocariosa ATCC 17931	171
Shinella fusca	4467
Skermanella aerolata	2
Snodgrassella alvi wkB2	16055
Sphingomonas echinoides	8777

Staphylococcus epidermidis	11709
Streptococcus oralis subsp. dentisani	9801
Streptococcus salivarius	19617
Unassigned	12379

Table S8: Total taxonomic distribution of active ASVs in midgut exposed at 1 ppb

ASV Species	ASV activity
Actinomyces johnsonii	3
Bacillus wiedmannii	2
Bacteroides caccae	1
Bacteroides dorei	4
Bartonella apis	2029
Bifidobacterium asteroides	33
Bifidobacterium callitrichos DSM 23973	6
Bifidobacterium coryneforme	111
Blautia wexlerae DSM 19850	1
Bombella apis	118
Bradyrhizobium oligotrophicum S58	8
Bradyrhizobium vignae	6
Dermacoccus profundi	6
Escherichia fergusonii ATCC 35469	4
Faecalibacterium prausnitzii	1
Flavobacterium acidificum	15
Frischella perrara	1931
Gemmiger formicilis	1

Gilliamella apicola	2077
Intestinibacter bartlettii	2
Lactobacillus apis	10196
Lactobacillus helsingborgensis	1562
Lactobacillus kimbladii	132
Lactobacillus kullabergensis	16
Lactobacillus kunkeei	31
Lactobacillus mellifer	28
Lactobacillus mellis	303
Lactobacillus melliventris	209
Leifsonia shinshuensis	5
Lysinibacillus parviboronicapiens	9
Methylorubrum populi BJ001	1
Oceanobacillus chironomi	3
Parasaccharibacter apium	275
Pediococcus stilesii	2
Pelomonas saccharophila	73
Phascolarctobacterium faecium	7
Pseudaminobacter manganicus	26
Pseudomonas canadensis	180
Pseudomonas cerasi	67
Pseudomonas songnenensis	30
Pseudoxanthomonas jiangsuensis	86
Psychrobacter cryohalolentis K5	294
Ralstonia insidiosa	369
Ralstonia pickettii	322

Romboutsia timonensis	1226
Roseburia faecis	1137
Rothia dentocariosa ATCC 17931	164
Shinella fusca	6860
Skermanella aerolata	6777
Snodgrassella alvi wkB2	15599
Sphingomonas echinoides	13694
Staphylococcus epidermidis	8321
Streptococcus oralis subsp. dentisani	1493
Streptococcus salivarius	23362
Unassigned	13742

Table S9: Total taxonomic distribution of active ASVs in midgut exposed at 10 ppb

ASV Species	ASV activity
Achromobacter insolitus	4
Actinomyces johnsonii	1
Bacillus wiedmannii	54
Bartonella apis	5560
Bifidobacterium asteroides	139
Bifidobacterium callitrichos DSM 23973	6
Bifidobacterium coryneforme	138
Bombella apis	2
Bradyrhizobium vignae	2
Delftia acidovorans	5
Dermacoccus profundi	11

Faecalibacterium prausnitzii	2
Frischella perrara	7093
Gilliamella apicola	16389
Gilliamella bombicola	4
Gilliamella intestini	5
Intestinibacter bartlettii	2
Lactobacillus apis	24330
Lactobacillus helsingborgensis	3054
Lactobacillus kimbladii	1081
Lactobacillus kullabergensis	244
Lactobacillus kunkeei	9
Lactobacillus mellifer	112
Lactobacillus mellis	760
Lactobacillus melliventris	1753
Lawsonella clevelandensis	4
Leifsonia shinshuensis	1
Parasaccharibacter apium	1161
Pediococcus stilesii	24
Pelomonas saccharophila	117
Pseudaminobacter manganicus	102
Pseudomonas canadensis	95
Pseudomonas cerasi	191
Pseudomonas songnenensis	22
Pseudoxanthomonas jiangsuensis	8
Psychrobacter cryohalolentis K5	185
Ralstonia insidiosa	9

Ralstonia pickettii	168
Romboutsia timonensis	772
Roseburia faecis	1487
Rothia dentocariosa ATCC 17931	1
Shinella fusca	2589
Skermanella aerolata	1159
Snodgrassella alvi wkB2	10116
Sphingomonas echinoides	3775
Staphylococcus epidermidis	9959
Streptococcus oralis subsp. dentisani	3399
Streptococcus salivarius	10126
Unassigned	14842

Table S10 : Total taxonomic distribution of active ASVs in ileum exposed at 0 ppb

**ASV Species**	**ASV activity**
Achromobacter insolitus	3
Acinetobacter defluvii	2
Arthrobacter russicus	5
Bacillus wiedmannii	1
Bacteroides caccae	3
Bartonella apis	5211
Bifidobacterium asteroides	10
Bifidobacterium callitrichos DSM 23973	1
Bifidobacterium coryneforme	48
Blautia wexlerae DSM 19850	21

Bombella apis	39
Bradyrhizobium vignae	4
Brevundimonas albigilva	4
Dermacoccus profundi	2
Escherichia fergusonii ATCC 35469	1
Faecalibacterium prausnitzii	3
Frischella perrara	23313
Gilliamella apicola	5795
Gilliamella bombicola	6
Gilliamella intestini	3
Lachnoclostridium pacaense	4
Lactobacillus apis	23973
Lactobacillus helsingborgensis	3255
Lactobacillus kimbladii	580
Lactobacillus kullabergensis	54
Lactobacillus kunkeei	3
Lactobacillus mellifer	98
Lactobacillus mellis	609
Lactobacillus melliventris	641
Lactobacillus rogosae	1
Lysinibacillus parviboronicapiens	4
Oceanobacillus chironomi	3
Parasaccharibacter apium	17
Pediococcus stilesii	159
Pelomonas saccharophila	21
Pseudaminobacter manganicus	1

Pseudomonas canadensis	82
Pseudomonas cerasi	35
Pseudomonas songnenensis	71
Pseudoxanthomonas jiangsuensis	5
Psychrobacter cryohalolentis K5	26
Ralstonia pickettii	428
Romboutsia timonensis	511
Roseburia faecis	437
Rothia dentocariosa ATCC 17931	85
Shinella fusca	2336
Skermanella aerolata	235
Snodgrassella alvi wkB2	19553
Sphingomonas echinoides	8825
Staphylococcus epidermidis	4802
Streptococcus oralis subsp. dentisani	1645
Streptococcus salivarius	12975
Unassigned	8837

Table S11 : Total taxonomic distribution of active ASVs in ileum exposed at 0.1 ppb

**ASV Species**	**ASV activity**
Achromobacter insolitus	3
Acinetobacter defluvii	6
Actinomyces johnsonii	1
Bacillus wiedmannii	14
Bacteroides caccae	2
Bacteroides dorei	2

Bartonella apis	7950
Bifidobacterium asteroides	15
Bifidobacterium callitrichos DSM 23973	3
Bifidobacterium coryneforme	110
Bradyrhizobium oligotrophicum S58	1
Bradyrhizobium vignae	10
Brevundimonas albigilva	2
Chryseobacterium hispanicum	1
Delftia acidovorans	8
Dermacoccus profundi	6
Flavobacterium acidificum	7
Frischella perrara	9153
Gilliamella apicola	5041
Gilliamella bombicola	4
Gilliamella intestini	11
Lachnoclostridium pacaense	4
Lactobacillus apis	29174
Lactobacillus helsingborgensis	4113
Lactobacillus kimbladii	446
Lactobacillus kullabergensis	80
Lactobacillus mellifer	83
Lactobacillus mellis	311
Lactobacillus melliventris	1320
Lawsonella clevelandensis	3
Leifsonia shinshuensis	1
Lysinibacillus parviboronicapiens	3

Methylorubrum populi BJ001	5
Morganella morganii subsp. sibonii	2
Parasaccharibacter apium	1
Pediococcus stilesii	216
Pelomonas saccharophila	23
Phascolarctobacterium faecium	122
Pseudaminobacter manganicus	11
Pseudomonas canadensis	95
Pseudomonas cerasi	131
Pseudomonas songnenensis	53
Pseudoxanthomonas jiangsuensis	252
Psychrobacter cryohalolentis K5	186
Ralstonia insidiosa	252
Ralstonia pickettii	886
Romboutsia timonensis	275
Roseburia faecis	430
Rothia dentocariosa ATCC 17931	2316
Shinella fusca	2918
Skermanella aerolata	64
Snodgrassella alvi wkB2	17322
Sphingomonas echinoides	6272
Staphylococcus epidermidis	5320
Streptococcus oralis subsp. dentisani	2809
Streptococcus salivarius	12297
Unassigned	9726

Table S12 : Total taxonomic distribution of active ASVs in ileum exposed at 1 ppb

ASV Species	ASV activity
[Clostridium] saccharolyticum	5
Achromobacter insolitus	6
Arthrobacter russicus	1
Bacillus wiedmannii	182
Bacteroides caccae	1
Bacteroides dorei	5
Bartonella apis	4793
Bifidobacterium asteroides	22
Bifidobacterium callitrichos DSM 23973	4
Bifidobacterium coryneforme	49
Bradyrhizobium oligotrophicum S58	7
Bradyrhizobium vignae	39
Brevundimonas albigilva	6
Brevundimonas lenta	9
Chryseobacterium hispanicum	6
Delftia acidovorans	10
Dermacoccus profundi	3
Dialister invisus	5
Escherichia fergusonii ATCC 35469	4
Flavobacterium acidificum	2
Frischella perrara	16368
Gemmiger formicilis	1
Gilliamella apicola	1903

Gilliamella bombicola	1
Lachnoclostridium pacaense	5
Lactobacillus apis	15168
Lactobacillus helsingborgensis	2745
Lactobacillus kimbladii	237
Lactobacillus kullabergensis	20
Lactobacillus kunkeei	15
Lactobacillus mellifer	34
Lactobacillus mellis	334
Lactobacillus melliventris	611
Lactobacillus rogosae	1
Lawsonella clevelandensis	2
Lysinibacillus parviboronicapiens	2
Methylorubrum populi BJ001	8
Morganella morganii subsp. sibonii	6
Oceanobacillus chironomi	2
Parasaccharibacter apium	339
Pediococcus stilesii	76
Pelomonas saccharophila	11
Phascolarctobacterium faecium	16
Phascolarctobacterium succinatutens	122
Pseudaminobacter manganicus	5
Pseudomonas canadensis	54
Pseudomonas cerasi	35
Pseudomonas songnenensis	20
Pseudoxanthomonas jiangsuensis	31

Psychrobacter cryohalolentis K5	95
Ralstonia insidiosa	15
Ralstonia pickettii	150
Romboutsia timonensis	450
Roseburia faecis	626
Shinella fusca	3512
Skermanella aerolata	163
Snodgrassella alvi wkB2	9250
Sphingomonas echinoides	16699
Staphylococcus epidermidis	6428
Streptococcus oralis subsp. dentisani	499
Streptococcus salivarius	18116
Unassigned	12014

Table S13 : Total taxonomic distribution of active ASVs in ileum exposed at 10 ppb

ASV Species	ASV activity
Bacillus wiedmannii	80
Bacteroides caccae	5
Bacteroides dorei	2
Bartonella apis	5718
Bifidobacterium asteroides	95
Bifidobacterium callitrichos DSM 23973	6
Bifidobacterium coryneforme	237
Bradyrhizobium vignae	2
Dermacoccus profundi	13
Escherichia fergusonii ATCC 35469	1

Flavobacterium acidificum	1
Frischella perrara	5725
Gilliamella apicola	9200
Gilliamella bombicola	17
Gilliamella intestini	29
Lachnoclostridium pacaense	19
Lactobacillus apis	27304
Lactobacillus helsingborgensis	2839
Lactobacillus kimbladii	869
Lactobacillus kullabergensis	176
Lactobacillus kunkeei	8
Lactobacillus mellifer	83
Lactobacillus mellis	643
Lactobacillus melliventris	1297
Lawsonella clevelandensis	5
Methylorubrum populi BJ001	3
Parasaccharibacter apium	37
Pediococcus stilesii	180
Pelomonas saccharophila	29
Phascolarctobacterium faecium	5
Pseudomonas canadensis	168
Pseudomonas cerasi	119
Pseudomonas songnenensis	39
Pseudoxanthomonas jiangsuensis	3
Psychrobacter cryohalolentis K5	103
Ralstonia insidiosa	7

Ralstonia pickettii	393
Romboutsia timonensis	271
Roseburia faecis	1225
Rothia dentocariosa ATCC 17931	4
Shinella fusca	2841
Skermanella aerolata	59
Snodgrassella alvi wkB2	14705
Sphingomonas echinoides	10557
Staphylococcus epidermidis	8745
Streptococcus oralis subsp. dentisani	7369
Streptococcus salivarius	17734
Unassigned	17326

Table S14 : Total taxonomic distribution of active ASVs in rectum exposed at 0 ppb

ASV Species	ASV activity
Arthrobacter russicus	3
Bacteroides caccae	1
Bacteroides plebeius DSM 17135	2
Bartonella apis	9066
Bifidobacterium asteroides	30
Bifidobacterium callitrichos DSM 23973	9
Bifidobacterium coryneforme	49
Bradyrhizobium vignae	1
Brevundimonas albigilva	2
Brevundimonas lenta	1
Chryseobacterium hispanicum	10

Delftia acidovorans	1
Dermacoccus profundi	11
Dialister invisus	1
Escherichia fergusonii ATCC 35469	13
Faecalibacterium prausnitzii	12
Flavobacterium acidificum	10
Frischella perrara	6051
Gemmiger formicilis	15
Gilliamella apicola	4582
Gilliamella bombicola	2
Lachnoclostridium pacaense	58
Lactobacillus apis	19221
Lactobacillus helsingborgensis	5750
Lactobacillus kimbladii	475
Lactobacillus kullabergensis	206
Lactobacillus kunkeei	8
Lactobacillus mellifer	66
Lactobacillus mellis	1447
Lactobacillus melliventris	1278
Lactobacillus rogosae	1
Lysinibacillus parviboronicapiens	3
Methylorubrum populi BJ001	3
Morganella morganii subsp. sibonii	1
Oceanobacillus chironomi	3
Parasaccharibacter apium	41
Pediococcus stilesii	2

Pelomonas saccharophila	51
Pseudaminobacter manganicus	19
Pseudomonas canadensis	21
Pseudomonas cerasi	152
Pseudomonas songnenensis	138
Pseudoxanthomonas jiangsuensis	46
Psychrobacter cryohalolentis K5	71
Ralstonia pickettii	827
Romboutsia timonensis	1015
Roseburia faecis	810
Rothia dentocariosa ATCC 17931	2121
Shinella fusca	7158
Skermanella aerolata	18
Snodgrassella alvi wkB2	16394
Sphingomonas echinoides	4781
Staphylococcus epidermidis	9962
Streptococcus oralis subsp. dentisani	4892
Streptococcus salivarius	22122
Unassigned	13213

Table S15 : Total taxonomic distribution of active ASVs in rectum exposed at 0.1 ppb

**ASV Species**	**ASV activity**
Achromobacter insolitus	1
Bacillus licheniformis	2
Bacillus wiedmannii	1
Bacteroides dorei	3

Bartonella apis	4194
Bifidobacterium asteroides	22
Bifidobacterium coryneforme	181
Bosea vaviloviae	1
Dermacoccus profundi	16
Faecalibacterium prausnitzii	1
Frischella perrara	1336
Gemmiger formicilis	1
Gilliamella apicola	3217
Lactobacillus apis	28894
Lactobacillus helsingborgensis	3699
Lactobacillus kimbladii	642
Lactobacillus kullabergensis	43
Lactobacillus kunkeei	4
Lactobacillus mellifer	35
Lactobacillus mellis	314
Lactobacillus melliventris	1990
Lawsonella clevelandensis	5
Lysinibacillus parviboronicapiens	2
Morganella morganii subsp. sibonii	2
Oceanobacillus chironomi	2
Pediococcus stilesii	3
Pelomonas saccharophila	86
Pseudaminobacter manganicus	31
Pseudomonas canadensis	2
Pseudomonas cerasi	99

Pseudomonas songnenensis	299
Pseudoxanthomonas jiangsuensis	7
Psychrobacter cryohalolentis K5	247
Ralstonia insidiosa	16
Ralstonia pickettii	685
Romboutsia timonensis	670
Roseburia faecis	948
Rothia dentocariosa ATCC 17931	14
Shinella fusca	6266
Snodgrassella alvi wkB2	15445
Sphingomonas echinoides	7055
Staphylococcus epidermidis	10553
Streptococcus oralis subsp. dentisani	5059
Streptococcus salivarius	16578
Unassigned	21276

Table S16 : Total taxonomic distribution of active ASVs in rectum exposed at 1 ppb

**ASV Species**	**ASV activity**
[Clostridium] saccharolyticum	3
Bacteroides caccae	1
Bartonella apis	7534
Bifidobacterium asteroides	21
Bifidobacterium callitrichos DSM 23973	7
Bifidobacterium coryneforme	62
Blautia wexlerae DSM 19850	1
Bradyrhizobium vignae	2

Dermacoccus profundi	14
Frischella perrara	4818
Gilliamella apicola	2004
Gilliamella bombicola	3
Gilliamella intestini	1
Lachnoclostridium pacaense	41
Lactobacillus apis	25179
Lactobacillus helsingborgensis	3021
Lactobacillus kimbladii	569
Lactobacillus kullabergensis	59
Lactobacillus kunkeei	12
Lactobacillus mellifer	115
Lactobacillus mellis	677
Lactobacillus melliventris	776
Lactobacillus rogosae	1
Lysinibacillus parviboronicapiens	8
Morganella morganii subsp. sibonii	1
Oceanobacillus chironomi	1
Pediococcus stilesii	6
Pelomonas saccharophila	38
Pseudaminobacter manganicus	36
Pseudomonas canadensis	5
Pseudomonas cerasi	113
Pseudomonas songnenensis	54
Pseudoxanthomonas jiangsuensis	3
Psychrobacter cryohalolentis K5	106

Ralstonia pickettii	434
Romboutsia timonensis	1316
Roseburia faecis	1781
Shinella fusca	4981
Skermanella aerolata	5
Snodgrassella alvi wkB2	17562
Sphingomonas echinoides	6088
Staphylococcus epidermidis	5581
Streptococcus oralis subsp. dentisani	1182
Streptococcus salivarius	18194
Unassigned	19626

Table S17 : Total taxonomic distribution of active ASVs in rectum exposed at 10 ppb

**ASV Species**	**ASV activity**
[Clostridium] saccharolyticum	3
Bacteroides caccae	1
Bartonella apis	7534
Bifidobacterium asteroides	21
Bifidobacterium callitrichos DSM 23973	7
Bifidobacterium coryneforme	62
Blautia wexlerae DSM 19850	1
Bradyrhizobium vignae	2
Dermacoccus profundi	14
Frischella perrara	4818
Gilliamella apicola	2004
Gilliamella bombicola	3

Gilliamella intestini	1
Lachnoclostridium pacaense	41
Lactobacillus apis	25179
Lactobacillus helsingborgensis	3021
Lactobacillus kimbladii	569
Lactobacillus kullabergensis	59
Lactobacillus kunkeei	12
Lactobacillus mellifer	115
Lactobacillus mellis	677
Lactobacillus melliventris	776
Lactobacillus rogosae	1
Lysinibacillus parviboronicapiens	8
Morganella morganii subsp. sibonii	1
Oceanobacillus chironomi	1
Pediococcus stilesii	6
Pelomonas saccharophila	38
Pseudaminobacter manganicus	36
Pseudomonas canadensis	5
Pseudomonas cerasi	113
Pseudomonas songnenensis	54
Pseudoxanthomonas jiangsuensis	3
Psychrobacter cryohalolentis K5	106
Ralstonia pickettii	434
Romboutsia timonensis	1316
Roseburia faecis	1781
Shinella fusca	4981

Skermanella aerolata	5
Snodgrassella alvi wkB2	17562
Sphingomonas echinoides	6088
Staphylococcus epidermidis	5581
Streptococcus oralis subsp. dentisani	1182
Streptococcus salivarius	18194
Unassigned	19626

Table S18 : Network metrics for gut microbiome taxa in midgut exposed to 0 ppb clothianidin

CC = Closeness centrality, DG = Degree, NC = Neighborhood Connectivity

Taxa name (spp.)	ASV classification	CC	DG	NC	ASV activity
Bartonella	Non-core	0.65671642	22	14.22727273	15007
Pediococcus	Low abundant	0.61971831	22	12.04545455	230
Pelomonas	Low abundant	0.61111111	17	16.41176471	1
Acinetobacter	Low abundant	0.61111111	17	16.41176471	4
Leifsonia	Low abundant	0.61111111	17	16.41176471	6
Morganella	Low abundant	0.61111111	17	16.41176471	2
Phascolarctobacterium	Low abundant	0.61111111	17	16.41176471	10
Staphylococcus	Low abundant	0.61111111	17	15.64705882	8
Devosia	Low abundant	0.61111111	17	16.41176471	17
Snodgrassella	Core	0.60273973	18	14.61111111	10367
Bradyrhizobium	Low abundant	0.58666667	15	16.13333333	43
Lachnoclostridium	Low abundant	0.57894737	16	14.375	155

Rothia	Low abundant	0.57142857	13	13.38461538	1
Streptococcus	Low abundant	0.57142857	13	13.38461538	36
Intestinibacter	Low abundant	0.57142857	13	13.38461538	63
Escherichia	Low abundant	0.56410256	12	11.41666667	1
Unassigned	unassigned	0.55696203	12	12.5	14420
Flavobacterium	Low abundant	0.55	16	14.375	10
Achromobacter	Low abundant	0.55	12	11.41666667	42
Prevotella	Low abundant	0.55	16	14.375	32
Frischella	Non-core	0.55	12	12.75	29145
Arthrobacter	Low abundant	0.54320988	15	15.33333333	29
Bosea	Low abundant	0.54320988	15	15.33333333	37
Lactobacillus	Core	0.54320988	12	13.08333333	38528
Pseudoxanthomonas	Low abundant	0.53012048	11	15.36363636	6
Brevundimonas	Low abundant	0.53012048	13	12.92307692	65
Delftia	Low abundant	0.53012048	10	12.4	96
Bifidobacterium	Core	0.51764706	11	10.36363636	195
Bombella	Non-core	0.51764706	7	13.28571429	204
Acidocella	Low abundant	0.51162791	8	12	10
Barnesiella	Low abundant	0.50574713	10	13	4
Dialister	Low abundant	0.50574713	10	13	7
Commensalibacter	Non-core	0.50574713	8	10	28
Roseburia	Low abundant	0.50574713	9	12.77777778	85
Pseudomonas	Low abundant	0.50574713	10	13	1
Actinomyces	Low abundant	0.49438202	6	13	6
Gilliamella	Non-core	0.49438202	9	12.77777778	11407

Gemmiger	Low abundant	0.48888889	6	11	1
Anaerobiospirillum	Low abundant	0.48888889	6	11	1
Phyllobacterium	Low abundant	0.48888889	6	11	2
Shinella	Low abundant	0.48351648	11	12.72727273	3
Parasaccharibacter	Non-core	0.46808511	10	11.1	1117
Faecalibacterium	Low abundant	0.44897959	8	11.5	6
Dermacoccus	Low abundant	0.44444444	3	14	2
Pseudaminobacter	Low abundant	0.40366972	3	9.33333333	2

Table S19 : Network metrics for gut microbiome taxa in midgut exposed to 0.1 ppb clothianidin

CC = Closeness centrality, DG = Degree, NC = Neighborhood Connectivity

Taxa name (spp.)	ASV Classification	CC	DG	NC	ASV activity
Frischella	Non-core	0.56666667	8	10.625	20817
Bartonella	Non-core	0.61818182	14	10.42857143	17761
Lactobacillus	Core	0.66666667	17	9.70588235	34363
Snodgrassella	Core	0.59649123	11	10.45454545	12928
Unassigned	unassigned	0.60714286	12	10.5	20940
Gilliamella	Non-core	0.53125	8	9.875	12598
Parasaccharibacter	Non-core	0.60714286	12	10.25	3
Bombella	Non-core	0.5862069	11	10.36363636	404
Bacillus	Low abundant	0.56666667	10	10.1	266
Bifidobacterium	Core	0.5483871	8	11.125	544
Pediococcus	Low abundant	0.5862069	11	10.54545455	256
Lachnoclostridium	Low abundant	0.60714286	13	10	31

Ralstonia	Low abundant	0.60714286	13	10.61538462	122
Pseudomonas	Low abundant	0.55737705	9	9.66666667	4
Delftia	Low abundant	0.53968254	7	9.85714286	6
Bradyrhizobium	Low abundant	0.51515152	7	10.85714286	17
Streptococcus	Low abundant	0.5862069	10	9.9	1
Commensalibacter	Non-core	0.61818182	13	9.92307692	72
Prevotella	Low abundant	0.5862069	10	12.2	1
Methylorubrum	Low abundant	0.5862069	11	10.63636364	3
Oceanobacillus	Low abundant	0.5862069	10	12.2	22
Lysinibacillus	Low abundant	0.52307692	7	11.42857143	1
Blautia	Low abundant	0.5483871	8	10.5	3
Faecalibacterium	Low abundant	0.5483871	8	10.5	1
Acidocella	Low abundant	0.55737705	8	11.5	26
Achromobacter	Low abundant	0.53125	7	10.42857143	2
Anaerobiospirillum	Low abundant	0.55737705	9	9.33333333	4
Staphylococcus	Low abundant	0.55737705	11	10	4
Shinella	Low abundant	0.53968254	10	9.7	12
Flavobacterium	Low abundant	0.5483871	10	10.4	3
Escherichia	Low abundant	0.5483871	8	10.625	1
Thiohalobacter	Low abundant	0.56666667	9	11.33333333	10
Acinetobacter	Low abundant	0.53125	7	10.42857143	1
Pelomonas	Low abundant	0.61818182	13	10.46153846	3
Moraxella	Low abundant	0.48571429	4	13.5	1

Table S20 : Network metrics for gut microbiome taxa in midgut exposed to 1 ppb clothianidin

CC = Closeness centrality, DG = Degree, NC = Neighborhood Connectivity

**Taxa name (spp.)**	**ASV Classification**	**CC**	**DG**	**NC**	**ASV activity**
Frischella	Non-core	0.66037736	18	9.77777778	11964
Lactobacillus	Core	0.57377049	11	10.90909091	49115
Gilliamella	Non-core	0.55555556	9	10.11111111	3576
Unassigned	unassigned	0.64814815	16	10.125	20649
Snodgrassella	Core	0.5	6	9.66666667	19115
Parasaccharibacter	Non-core	0.57377049	11	10.54545455	7050
Bombella	Non-core	0.58333333	11	10.54545455	282
Bacillus	Low abundant	0.47297297	7	9.71428571	372
Bifidobacterium	Core	0.56451613	13	9.84615385	522
Pediococcus	Low abundant	0.55555556	10	10.6	182
Lachnoclostridium	Low abundant	0.46666667	5	10	41
Ralstonia	Low abundant	0.57377049	11	9.45454545	10
Bartonella	Non-core	0.43209877	3	9.66666667	10349
Roseburia	Low abundant	0.55555556	10	10.4	2
Delftia	Low abundant	0.45454545	6	8.5	3
Bradyrhizobium	Low abundant	0.5	8	10.625	12
Streptococcus	Low abundant	0.56451613	11	10.90909091	5
Commensalibacter	Non-core	0.55555556	10	10.4	98
Bacteroides	Low abundant	0.58333333	10	9.8	5
Pseudoxanthomonas	Low abundant	0.53846154	8	8.875	6
Prevotella	Low abundant	0.56451613	10	9.5	1

Methylorubrum	Low abundant	0.546875	9	10.77777778	6
Phyllobacterium	Low abundant	0.53030303	11	9	32
Lysinibacillus	Low abundant	0.53030303	9	10.33333333	30
Faecalibacterium	Low abundant	0.33653846	1	6	4
Acidocella	Low abundant	0.53030303	9	10	46
Achromobacter	Low abundant	0.52238806	9	10.44444444	2
Anaerobiospirillum	Low abundant	0.42682927	4	8.75	5
Shinella	Low abundant	0.50724638	6	11.16666667	7
Flavobacterium	Low abundant	0.58333333	10	10.6	3
Dermacoccus	Low abundant	0.52238806	9	10.44444444	6
Chryseobacterium	Low abundant	0.5	7	10.14285714	1
Escherichia	Low abundant	0.58333333	11	10.72727273	3
Lawsonella	Low abundant	0.52238806	9	10.44444444	1
Porphyromonas	Low abundant	0.5	7	10.14285714	2
Mycobacterium	Low abundant	0.47297297	7	8.42857143	3

Table S21 : Network metrics for gut microbiome taxa in midgut exposed to 10 ppb clothianidin

CC = Closeness centrality, DG = Degree, NC = Neighborhood Connectivity

**Taxa name (spp.)**	**ASV Classification**	**CC**	**DG**	**NC**	**ASV activity**
Snodgrassella	Core	0.62745098	13	8.38461538	7386

Bifidobacterium	Core	0.60377358	14	9.5	595
Parasaccharibacter	Non-core	0.59259259	14	8.57142857	2317
Acidocella	Low abundant	0.59259259	10	9.4	58
Anaerobiospirillum	Low abundant	0.59259259	12	8.41666667	45
Rothia	Low abundant	0.59259259	11	9.90909091	1
Bartonella	Non-core	0.58181818	10	10.6	15516
Leifsonia	Low abundant	0.58181818	10	9.5	1
Thiohalobacter	Low abundant	0.58181818	10	9.5	1
Pelomonas	Low abundant	0.58181818	10	9.5	1
Commensalibacter	Non-core	0.57142857	9	10.1111111	111
Staphylococcus	Low abundant	0.56140351	9	9.66666667	3
Lactobacillus	Core	0.55172414	8	9.125	51835
Unassigned	unassigned	0.55172414	9	9.88888889	28981
Roseburia	Low abundant	0.55172414	8	10	3
Shinella	Low abundant	0.55172414	8	7.625	14
Bombella	Non-core	0.54237288	10	10.4	3
Achromobacter	Low abundant	0.54237288	10	10.4	6
Ralstonia	Low abundant	0.53333333	10	9.1	9
Pediococcus	Low abundant	0.49230769	6	8.83333333	121
Streptococcus	Low abundant	0.49230769	8	10.125	4
Phyllobacterium	Low abundant	0.49230769	8	10.125	3
Lawsonella	Low abundant	0.49230769	8	10.125	4
Actinomyces	Low abundant	0.49230769	8	10.125	1
Bacillus	Low abundant	0.48484848	7	8.28571429	54

Frischella	Non-core	0.48484848	7	8.57142857	10132
Pseudomonas	Low abundant	0.48484848	7	8	3
Lachnoclostridium	Low abundant	0.47761194	4	9.75	102
Flavobacterium	Low abundant	0.47761194	4	9.75	2
Gilliamella	Core	0.47058824	7	8.57142857	19862
Pseudoxanthomonas	Low abundant	0.45714286	4	8.5	1
Delftia	Low abundant	0.44444444	5	10.2	5
Faecalibacterium	Low abundant	0.4	2	7.5	3

Table S22 : Network metrics for gut microbiome taxa in ileum exposed to 0 ppb clothianidin

CC = Closeness centrality, DG = Degree, NC = Neighborhood Connectivity

Taxa name (spp.)	ASV Classification	CC	DG	NC	ASV activity
Bartonella	Non-core	0.62962963	19	11.10526316	10013
Lactobacillus	Core	0.61818182	13	10	48171
Commensalibacter	Non-core	0.59649123	16	10.875	48
Pediococcus	Low abundant	0.59649123	13	10.38461538	236
Bifidobacterium	Core	0.59649123	17	10.82352941	108
Unassigned	Unassigned	0.59649123	16	10.875	9830
Frischella	Low abundant	0.59649123	12	11.5	30344
Snodgrassella	Core	0.5862069	14	10.92857143	24749

Gilliamella	Core	0.57627119	12	9.25	7453
Lachnoclostridium	Low abundant	0.56666667	8	8.75	5
Blautia	Low abundant	0.55737705	9	12.22222222	21
Parasaccharibacter	Non-core	0.55737705	8	9.5	252
Sphingomonas	Low abundant	0.53968254	8	10.625	1
Brevundimonas	Low abundant	0.53968254	12	11.75	4
Acinetobacter	Low abundant	0.53125	11	13.81818182	2
Escherichia	Low abundant	0.53125	11	13.81818182	1
Faecalibacterium	Low abundant	0.53125	11	13.81818182	3
Bacteroides	Low abundant	0.53125	11	13.81818182	2
Achromobacter	Low abundant	0.52307692	7	11.42857143	1
Acidocella	Low abundant	0.51515152	8	12.125	10
Pelomonas	Low abundant	0.50746269	7	12	1
Arthrobacter	Low abundant	0.50746269	9	13	5
Shinella	Low abundant	0.5	7	13.71428571	2
Delftia	Low abundant	0.5	7	13.71428571	1
Bombella	Non-core	0.5	6	11.33333333	123
Oceanobacillus	Low abundant	0.47887324	6	11.33333333	2

Pseudaminobacter	Low abundant	0.45945946	4	10.25	3
Methylorubrum	Low abundant	0.45333333	4	7.75	25
Flavobacterium	Low abundant	0.44736842	3	9.66666667	3
Anaerobiospirillum	Low abundant	0.44155844	5	7	3
Rothia	Low abundant	0.41975309	3	7.33333333	2
Streptococcus	Low abundant	0.4047619	5	5.4	2
Roseburia	Low abundant	0.4047619	5	5.4	2
Pseudomonas	Low abundant	0.4047619	5	5.4	5
Bradyrhizobium	Low abundant	0.34693878	4	4.75	5

Table S23 : Network metrics for gut microbiome taxa in ileum exposed to 0.1 ppb clothianidin

CC = Closeness centrality, DG = Degree, NC = Neighborhood Connectivity

Taxa name (spp.)	ASV Classification	CC	DG	NC	ASV activity
Unassigned	unassigned	0.65079365	19	10.84210526	14500
Bifidobacterium	Core	0.63076923	17	9.64705882	318
Acidocella	Low abundant	0.60294118	14	12.35714286	13
Streptococcus	Low abundant	0.60294118	15	11.4	7
Bartonella	Non-core	0.60294118	15	11.8	13269

Actinomyces	Low abundant	0.57746479	12	11.16666667	1
Lawsonella	Low abundant	0.57746479	12	11.16666667	3
Pediococcus	Low abundant	0.57746479	12	11	283
Bacillus	Low abundant	0.57746479	14	10.64285714	116
Oceanobacillus	Low abundant	0.56944444	14	10.92857143	1
Phyllobacterium	Low abundant	0.56944444	14	10.92857143	3
Delftia	Low abundant	0.56944444	12	11.16666667	8
Pelomonas	Low abundant	0.56164384	10	12.6	2
Shinella	Low abundant	0.56164384	11	10.81818182	12
Acinetobacter	Low abundant	0.55405405	11	10.63636364	4
Flavobacterium	Low abundant	0.54666667	11	12.90909091	10
Lactobacillus	Core	0.54666667	12	11.66666667	55511
Sphingomonas	Low abundant	0.53947368	10	10.4	6
Ralstonia	Low abundant	0.53947368	11	8.54545455	285
Lachnoclostridium	Low abundant	0.53947368	8	13.875	12
Frischella	Non-core	0.53947368	11	9	15057
Polynucleobacter	Low abundant	0.53246753	7	13.57142857	4

Leifsonia	Low abundant	0.53246753	7	13.57142857	1
Commensalibacter	Non-core	0.53246753	10	10.3	44
Anaerobiospirillum	Low abundant	0.52564103	9	13.44444444	6
Rothia	Low abundant	0.51898734	8	12.875	1
Psychrobacter	Low abundant	0.51898734	8	9.5	18
Bradyrhizobium	Low abundant	0.5125	8	10.875	12
Roseburia	Low abundant	0.5125	7	11	2
Chryseobacterium	Low abundant	0.50617284	8	9.625	1
Achromobacter	Low abundant	0.50617284	9	10.44444444	3
Methylorubrum	Low abundant	0.50617284	8	9.625	1
Bombella	Non-core	0.50617284	7	11	2316
Gilliamella	Core	0.50617284	10	9.8	7918
Parasaccharibacter	Non-core	0.50617284	7	11	64
Thiohalobacter	Low abundant	0.5	6	11.33333333	2
Bacteroides	Low abundant	0.5	6	11	1
Snodgrassella	Core	0.5	7	11.14285714	20309
Staphylococcus	Low abundant	0.47674419	6	12.66666667	1
Lysinibacillus	Low abundant	0.47674419	6	12.66666667	2

Pseudomonas	Low abundant	0.46590909	8	10	122
Brevundimonas	Low abundant	0.46067416	5	9.8	4

Table S24 : Network metrics for gut microbiome taxa in ileum exposed to 1 ppb clothianidin

CC = Closeness centrality, DG = Degree, NC = Neighborhood Connectivity

Taxa name (spp.)	ASV Classification	CC	DG	NC	ASV activity
Methylorubrum	Low abundant	0.68421053	22	14.31818182	10
Bifidobacterium	Core	0.67241379	20	13.5	177
Flavobacterium	Low abundant	0.66101695	19	15.15789474	7
Morganella	Low abundant	0.66101695	19	15.15789474	12
Staphylococcus	Low abundant	0.63934426	18	14.44444444	18
Sphingomonas	Low abundant	0.61904762	17	17.17647059	1
Escherichia	Low abundant	0.61904762	17	17.17647059	4
Chryseobacterium	Low abundant	0.61904762	17	17.17647059	6
Psychrobacter	Low abundant	0.61904762	17	17.17647059	4
Achromobacter	Low abundant	0.61904762	17	17.17647059	6
Arthrobacter	Low abundant	0.61904762	17	17.17647059	1
Pseudoxanthomonas	Low abundant	0.61904762	17	17.17647059	29
Ralstonia	Low abundant	0.61904762	18	14.05555556	34
Bradyrhizobium	Low abundant	0.609375	16	15.3125	42
Pediococcus	Low abundant	0.609375	17	16	126

Bartonella	Non-core	0.609375	14	11.14285714	11207
Frischella	Non-core	0.609375	15	12.86666667	25966
Pseudomonas	Low abundant	0.6	14	14.64285714	12
Commensalibacter	Non-core	0.59090909	14	15.64285714	96
Acidocella	Low abundant	0.58208955	12	12.91666667	20
Lawsonella	Low abundant	0.57352941	11	14.81818182	1
Romboutsia	Low abundant	0.57352941	11	14.81818182	122
Moraxella	Low abundant	0.55714286	11	13.45454545	2
Pelomonas	Low abundant	0.55714286	11	13.45454545	1
Shinella	Low abundant	0.55714286	9	13.11111111	7
Prevotella	Low abundant	0.55714286	11	13.45454545	2
Anaerobiospirillum	Low abundant	0.54929577	10	13.8	9
Parasaccharibacter	Non-core	0.54929577	11	13.54545455	502
Unassigned	unassigned	0.54166667	13	13.84615385	14462
Delftia	Low abundant	0.54166667	7	9.57142857	12
Lachnoclostridium	Low abundant	0.53424658	8	15.375	13
Snodgrassella	Core	0.52702703	9	12.66666667	21301

Gilliamella	Core	0.52	8	11.125	2408
Gemmiger	Low abundant	0.51315789	6	10.16666667	1
Streptococcus	Low abundant	0.51315789	9	13.44444444	9
Brevundimonas	Low abundant	0.50649351	5	10.6	9
Lysinibacillus	Low abundant	0.49367089	5	15.6	16
Bacillus	Low abundant	0.4875	5	9.4	196
Lactobacillus	Core	0.46428571	6	8.16666667	44837
Thiohalobacter	Low abundant	0.42391304	3	10	1

Table S25 : Network metrics for gut microbiome taxa in ileum exposed to 10 ppb clothianidin

CC = Closeness centrality, DG = Degree, NC = Neighborhood Connectivity

Taxa name (spp.)	ASV Classification	CC	DG	NC	ASV activity
Frischella	Non-core	0.58	11	6.72727273	16636
Blautia	Low abundant	0.56862745	9	6.33333333	1
Lactobacillus	Core	0.54716981	7	7.71428571	61901
Pediococcus	Low abundant	0.53703704	10	7	356
Bombella	Non-core	0.53703704	7	6.28571429	1
Snodgrassella	Core	0.53703704	7	7.85714286	20535

Commensalibacter	Non-core	0.52727273	6	7.16666667	115
Gilliamella	Core	0.52727273	7	6.85714286	16658
Bifidobacterium	Core	0.51785714	8	6.25	474
Unassigned	unassigned	0.51785714	8	6.125	24882
Thiohalobacter	Low abundant	0.50877193	6	6.66666667	1
Acidocella	Low abundant	0.5	5	7.6	31
Rothia	Low abundant	0.49152542	8	8.625	3
Escherichia	Low abundant	0.49152542	8	8.625	1
Skermanella	Low abundant	0.49152542	8	8.625	20
Bradyrhizobium	Low abundant	0.49152542	8	8.625	1
Pseudomonas	Low abundant	0.49152542	8	8.625	1
Pelomonas	Low abundant	0.48333333	6	7.16666667	10
Staphylococcus	Low abundant	0.47540984	8	7.875	4
Methylorubrum	Low abundant	0.47540984	8	7.875	3
Ralstonia	Low abundant	0.47540984	7	6.14285714	11
Roseburia	Low abundant	0.43939394	4	6	2
Bartonella	Non-core	0.43939394	4	7.25	14459

Bacillus	Low abundant	0.43283582	3	6	86
Flavobacterium	Low abundant	0.42647059	4	6.25	2
Anaerobiospirillum	Low abundant	0.42647059	4	6	32
Lachnoclostridium	Low abundant	0.41428571	3	6	20
Parasaccharibacter	Non-core	0.41428571	4	5	75
Shinella	Low abundant	0.40277778	3	6	20
Streptococcus	Low abundant	0.39189189	3	5.66666667	5

Table S26 : Network metrics for gut microbiome taxa in rectum exposed to 0 ppb clothianidin

CC = Closeness centrality, DG = Degree, NC = Neighborhood Connectivity

**Taxa name (spp.)**	**ASV Classification**	**CC**	**DG**	**NC**	**ASV activity**
Dialister	Low abundant	0.75	22	19.77272727	2
Morganella	Low abundant	0.75	22	19.77272727	1
Gemmiger	Low abundant	0.75	22	19.77272727	10
Phascolarctobacterium	Low abundant	0.75	22	19.77272727	4
Staphylococcus	Low abundant	0.75	22	19.77272727	1
Faecalibacterium	Low abundant	0.75	22	19.77272727	21

Blautia	Low abundant	0.75	22	19.77272727	11
Bacteroides	Low abundant	0.75	22	19.77272727	32
Streptococcus	Low abundant	0.75	22	19.77272727	3
Brevundimonas	Low abundant	0.75	22	19.77272727	1
Ralstonia	Low abundant	0.75	22	19.77272727	1
Acidocella	Low abundant	0.73333333	21	17.23809524	48
Roseburia	Low abundant	0.70212766	20	19.1	33
Prevotella	Low abundant	0.6875	19	18.52631579	3
Unassigned	unassigned	0.67346939	19	18.68421053	26229
Bartonella	Non-core	0.67346939	18	17.38888889	19028
Bifidobacterium	Core	0.66	18	17.61111111	199
Delftia	Low abundant	0.64705882	17	19.58823529	3
Snodgrassella	Core	0.64705882	16	19.75	9248
Escherichia	Low abundant	0.63461538	17	19.64705882	7
Commensalibacter	Non-core	0.63461538	18	18.72222222	59
Lachnoclostridium	Low abundant	0.63461538	17	18.52941176	81
Lactobacillus	Core	0.61111111	15	18.46666667	65178

Pediococcus	Low abundant	0.55	7	9	23
Anaerobiospirillum	Low abundant	0.54098361	6	16.16666667	12
Shinella	Low abundant	0.53225806	7	17.28571429	17
Bradyrhizobium	Low abundant	0.515625	6	10.16666667	3
Bombella	Non-core	0.515625	6	10.16666667	2121
Gilliamella	Core	0.50769231	5	13.4	9502
Flavobacterium	Low abundant	0.48529412	4	18.75	7
Frischella	Non-core	0.47826087	3	14.33333333	12408
Pelomonas	Low abundant	0.46478873	4	13.75	1
Acinetobacter	Low abundant	0.46478873	4	13.75	2
Parasaccharibacter	Non-core	0.44	3	9.33333333	28

Table S27 : Network metrics for gut microbiome taxa in rectum exposed to 0.1 ppb clothianidin

CC = Closeness centrality, DG = Degree, NC = Neighborhood Connectivity

Taxa name (spp.)	ASV Classification	CC	DG	NC	ASV activity
Bifidobacterium	Core	0.58536585	11	6.81818182	544
Bacillus	Low abundant	0.57142857	9	6.77777778	20
Pseudaminobacter	Non-core	0.55813953	7	7.14285714	1
Lysinibacillus	Low abundant	0.55813953	7	7.14285714	4
Frischella	Non-core	0.55813953	8	5	10075
Lactobacillus	Core	0.5106383	8	6.375	65373
Staphylococcus	Low abundant	0.5	5	7.6	1
Unassigned	unassigned	0.5	8	6.125	30672
Acidocella	Low abundant	0.48	6	7	50
Bartonella	Non-core	0.47058824	7	6	14746
Actinomyces	Low abundant	0.46153846	7	8	2
Leifsonia	Low abundant	0.46153846	7	8	1
Streptococcus	Low abundant	0.46153846	7	8	3
Bradyrhizobium	Low abundant	0.46153846	7	8	2
Brevundimonas	Low abundant	0.46153846	7	8	2

Lachnoclostridium	Low abundant	0.46153846	3	6.66666667	38
Snodgrassella	Core	0.46153846	8	6.875	13233
Shinella	Low abundant	0.45283019	4	7.5	24
Bacteroides	Low abundant	0.42857143	3	6	1
Commensalibacter	Non-core	0.42105263	4	6.5	107
Pediococcus	Low abundant	0.4	2	8	5
Gilliamella	Core	0.38709677	3	5.33333333	8282
Delftia	Low abundant	0.36923077	2	5	2
Bombella	Non-core	0.36923077	2	5	14
Anaerobiospirillum	Low abundant	0.35294118	2	3.5	20

Table S28 : Network metrics for gut microbiome taxa in rectum exposed to 1 ppb clothianidin

CC = Closeness centrality, DG = Degree, NC = Neighborhood Connectivity

Taxa name (spp.)	ASV Classification	CC	DG	NC	ASV activity
Lactobacillus	Core	0.64705882	11	6.90909091	63263
Commensalibacter	Non-core	0.62857143	10	7.9	167
Lachnoclostridium	Low abundant	0.61111111	8	7.375	79

Unassigned	unassigned	0.61111111	8	9	33295
Bartonella	Non-core	0.61111111	9	7	13115
Snodgrassella	Core	0.61111111	10	7.3	13936
Anaerobiospirillum	Low abundant	0.59459459	8	6.875	23
Acidocella	Low abundant	0.59459459	8	6.75	51
Escherichia	Low abundant	0.56410256	6	7.5	1
Oceanobacillus	Low abundant	0.56410256	6	7.5	1
Bifidobacterium	Core	0.55	8	7	239
Parasaccharibacter	Non-core	0.53658537	5	8	5
Pseudaminobacter	Low abundant	0.52380952	6	7.66666667	1
Morganella	Low abundant	0.52380952	6	7.66666667	1
Shinella	Low abundant	0.52380952	5	5.4	31
Bacteroides	Low abundant	0.51162791	5	7	2
Gilliamella	Core	0.51162791	6	6.5	3188
Lysinibacillus	Low abundant	0.5	5	6	15
Frischella	Non-core	0.48888889	5	7.6	11109
Blautia	Low abundant	0.47826087	3	9	1

Flavobacterium	Low abundant	0.45833333	4	6.25	2
Delftia	Low abundant	0.45833333	4	6.25	1
Pediococcus	Low abundant	0.41509434	2	8.5	11

Table S29 : Network metrics for gut microbiome taxa in rectum exposed to 10 ppb clothianidin

CC = Closeness centrality, DG = Degree, NC = Neighborhood Connectivity

Taxa name (spp.)	ASV Classification	CC	DG	NC	ASV activity
Snodgrassella	Core	0.67741935	12	7.66666667	7242
Bifidobacterium	Core	0.65625	11	8.54545455	642
Frischella	Non-core	0.65625	11	7.36363636	6984
Commensalibacter	Non-core	0.61764706	9	8.88888889	87
Bradyrhizobium	Low abundant	0.61764706	8	8.75	1
Unassigned	unassigned	0.61764706	9	8.44444444	26387
Acidocella	Low abundant	0.6	10	7.3	43
Shinella	Low abundant	0.56756757	7	9	15
Psychrobacter	Low abundant	0.56756757	6	7.5	1
Staphylococcus	Low abundant	0.55263158	7	9.71428571	1

Faecalibacterium	Low abundant	0.55263158	7	9.71428571	1
Pediococcus	Low abundant	0.55263158	8	7.5	7
Bacillus	Low abundant	0.55263158	5	7.2	1
Lachnoclostridium	Low abundant	0.53846154	7	6.42857143	59
Anaerobiospirillum	Low abundant	0.525	6	5.16666667	18
Bartonella	Non-core	0.525	5	7.6	11244
Gilliamella	Core	0.51219512	6	5.83333333	12959
Pseudaminobacter	Low abundant	0.5	5	9.2	1
Streptococcus	Low abundant	0.5	5	9.2	2
Lactobacillus	Core	0.47727273	3	7.33333333	58212
Parasaccharibacter	Non-core	0.44680851	2	7	50
Flavobacterium	Low abundant	0.41176471	3	6.33333333	1

Table S30 : Number of positive (+) and/or negative (-) interactions at the genus level in the microbial network depending on each honeybee gut section (midgut, ileum and rectum) and clothianidin concentrations (Ctrl (0 ppb), 0.1, 1 and 10 ppb)

Clothianidin Concentration	(+)			(-)		
	**Midgut**	**Ileum**	**Rectum**	**Midgut**	**Ileum**	**Rectum**
**Control (0 ppb)**	96	27	108	1	6	5
**0.1 ppb**	28	52	21	5	5	1
**1 ppb**	30	73	13	5	4	0
**10 ppb**	40	29	17	4	2	3

Figure S1 : Violin plot of Degree from taxa in midgut microbial networks

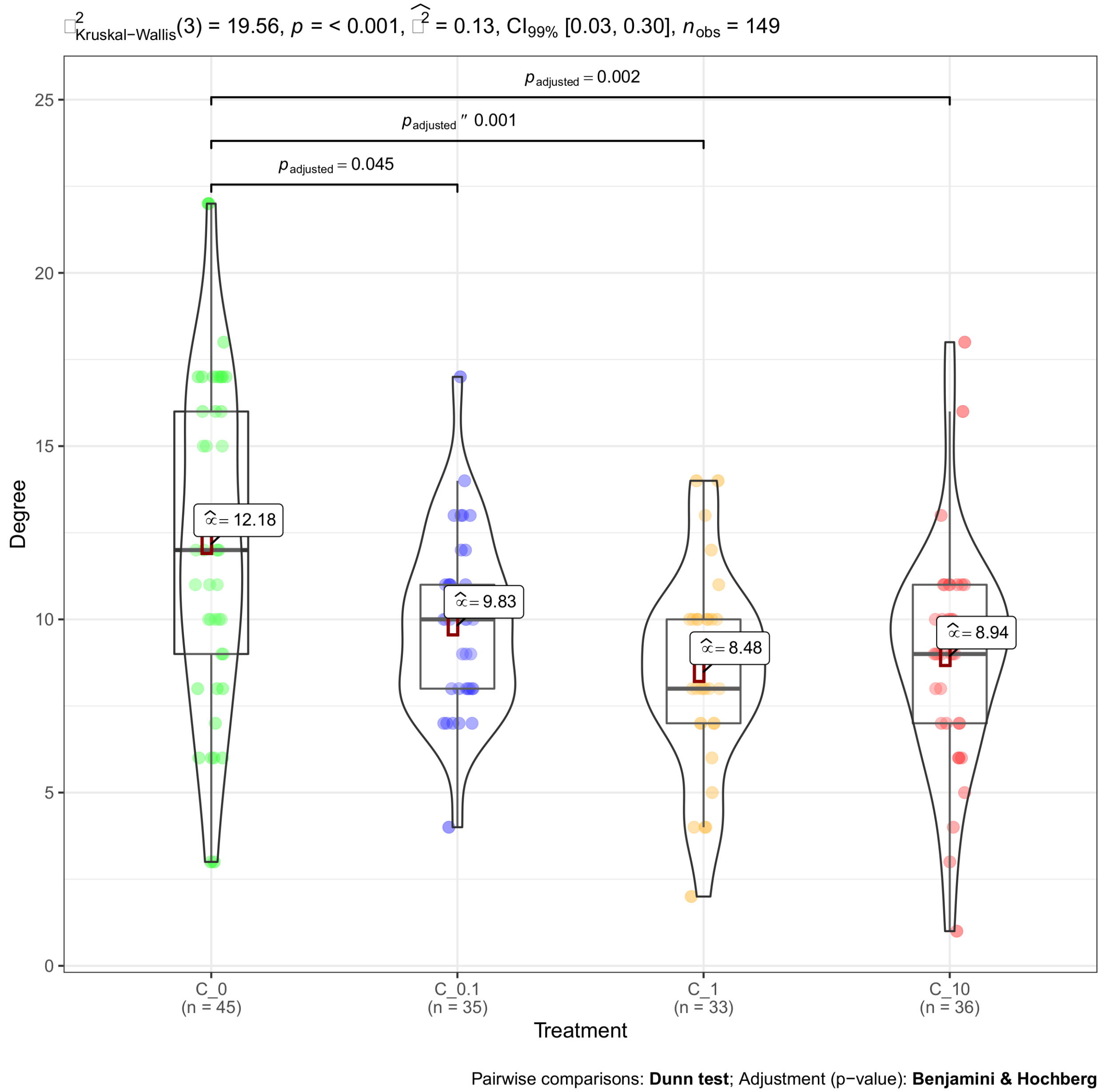

Figure S2 : Violin plot of Closeness Centrality from taxa in midgut microbial networks

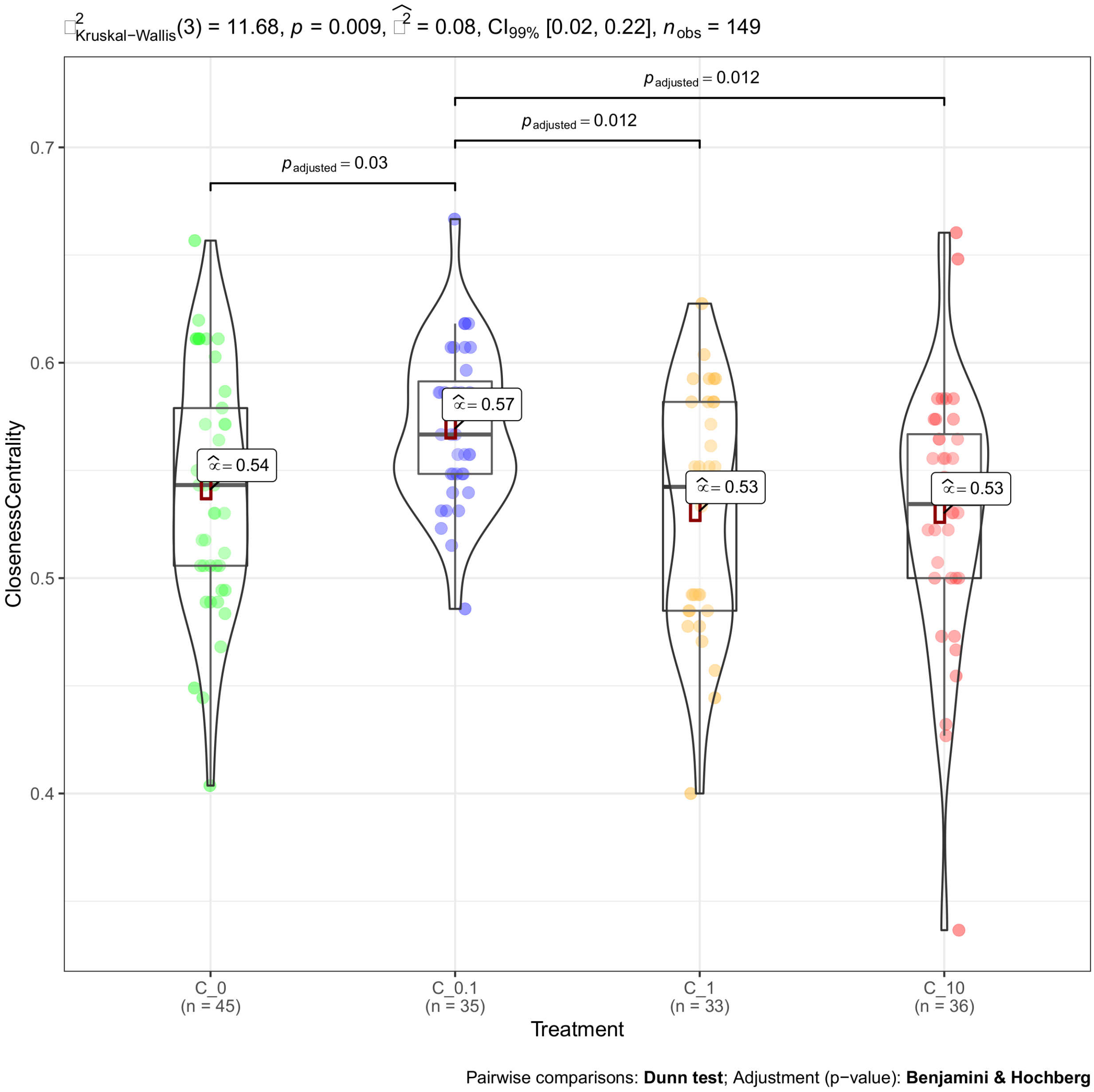

□²Kruskal−Wallis(3) = 11.68, p = 0.009, □̂² = 0.08, CI99% [0.02, 0.22], nobs = 149
padjusted = 0.012
padjusted = 0.012
padjusted = 0.03
0.7
0.6
0.5
0.4
ClosenessCentrality
μ̂ = 0.54
μ̂ = 0.57
μ̂ = 0.53
μ̂ = 0.53
C_0
(n = 45)
C_0.1
(n = 35)
C_1
(n = 33)
C_10
(n = 36)
Treatment
Pairwise comparisons: Dunn test; Adjustment (p−value): Benjamini & Hochberg

Figure S3 : Violin plot of Neighborhood Connectivity from taxa in midgut microbial networks

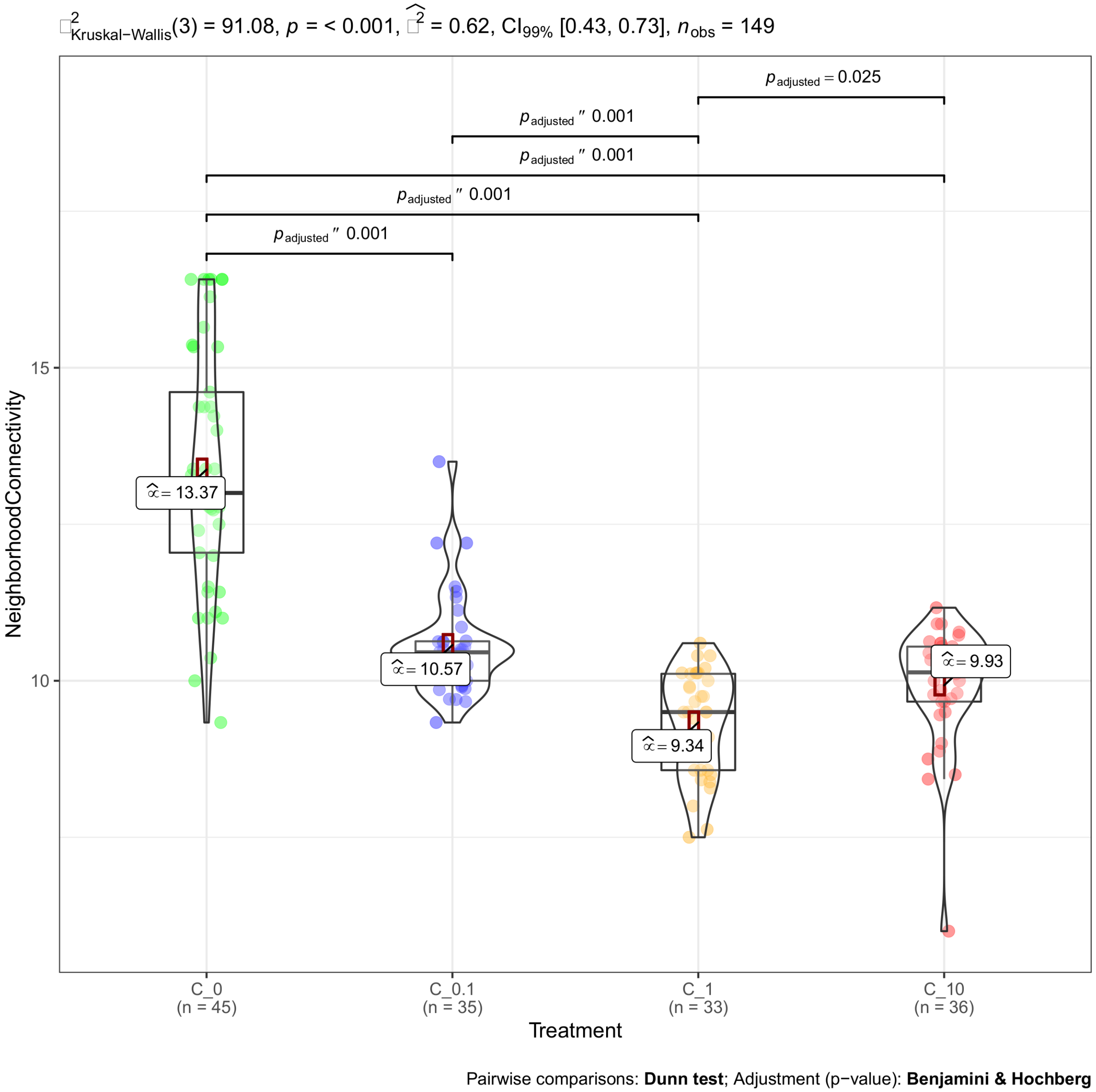

Figure S4 : Violin plot of Degree from taxa in ileum microbial networks

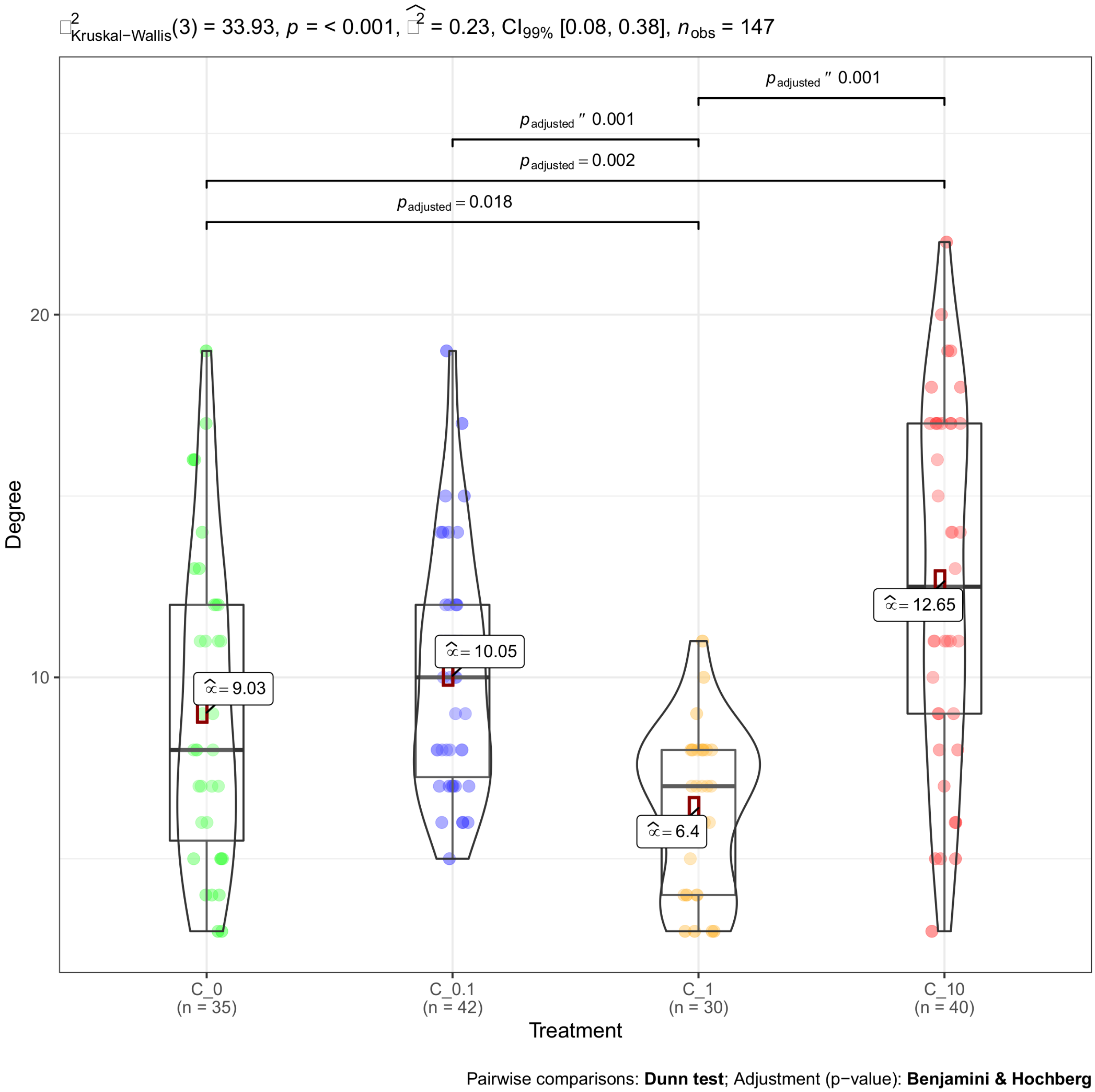

Figure S5 : Violin plot of Closeness Centrality from taxa in ileum microbial networks

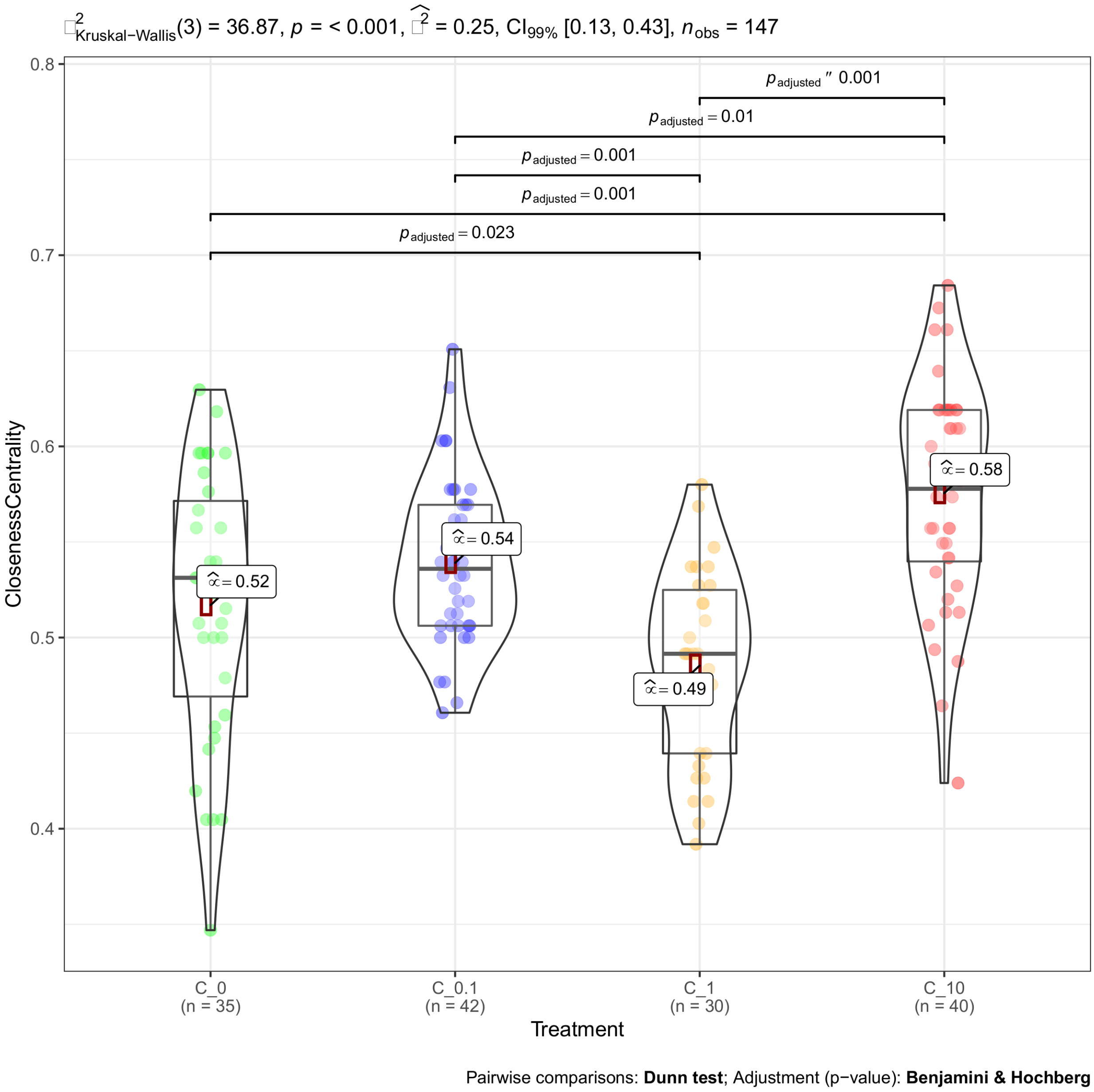

χ²Kruskal−Wallis(3) = 36.87, p = < 0.001, ε̂² = 0.25, CI99% [0.13, 0.43], nobs = 147
padjusted ≤ 0.001
padjusted = 0.01
padjusted = 0.001
padjusted = 0.001
padjusted = 0.023
ClosenessCentrality
0.8
0.7
0.6
0.5
0.4
μ̂ = 0.52
μ̂ = 0.54
μ̂ = 0.49
μ̂ = 0.58
C_0
(n = 35)
C_0.1
(n = 42)
C_1
(n = 30)
C_10
(n = 40)
Treatment
Pairwise comparisons: Dunn test; Adjustment (p−value): Benjamini & Hochberg

Figure S6 : Violin plot of Neighborhood Connectivity from taxa in ileum microbial networks

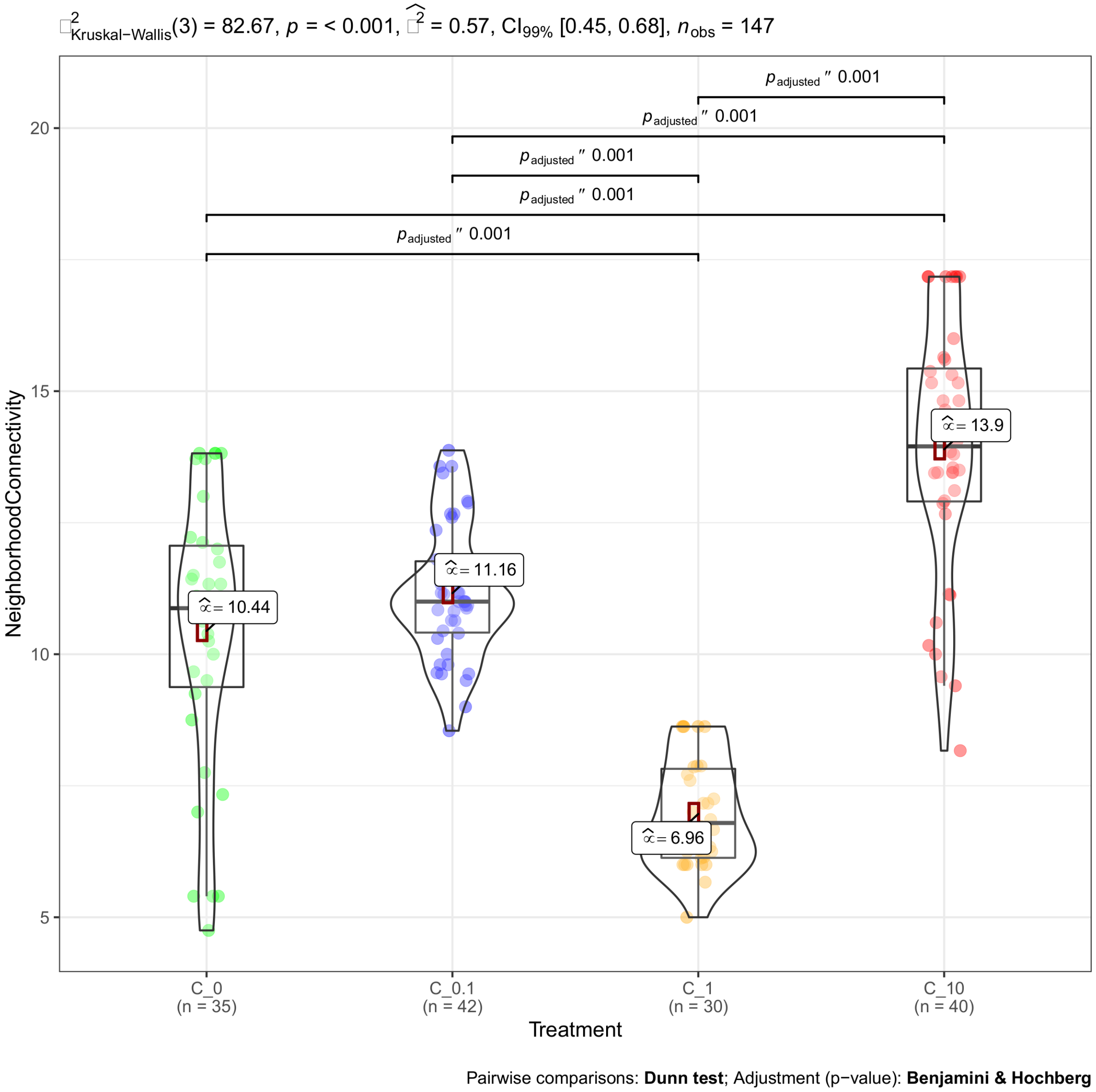

Figure S7 : Violin plot of Degree from taxa in rectum microbial networks

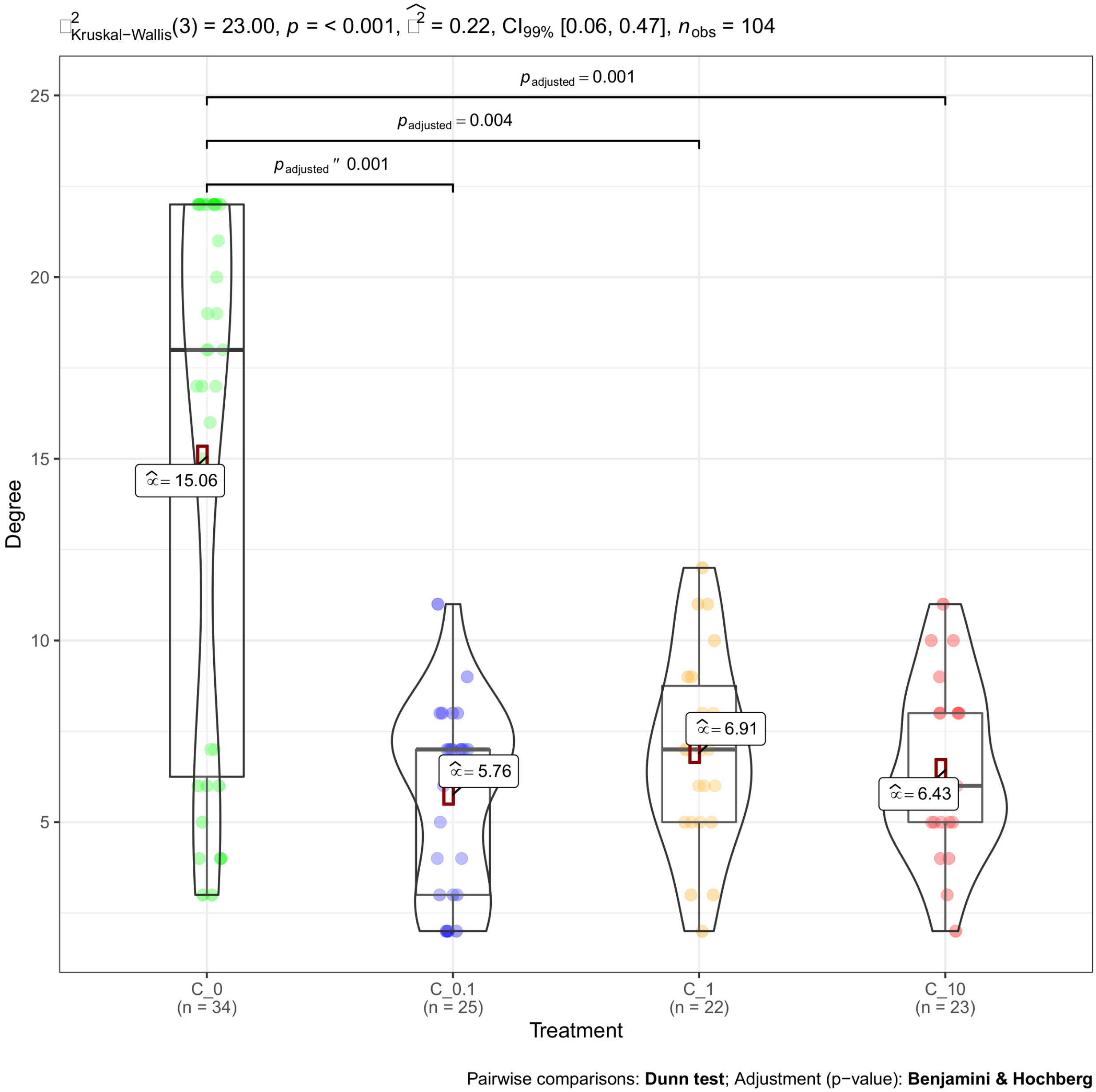

Figure S8 : Violin plot of Closeness Centrality from taxa in rectum microbial networks

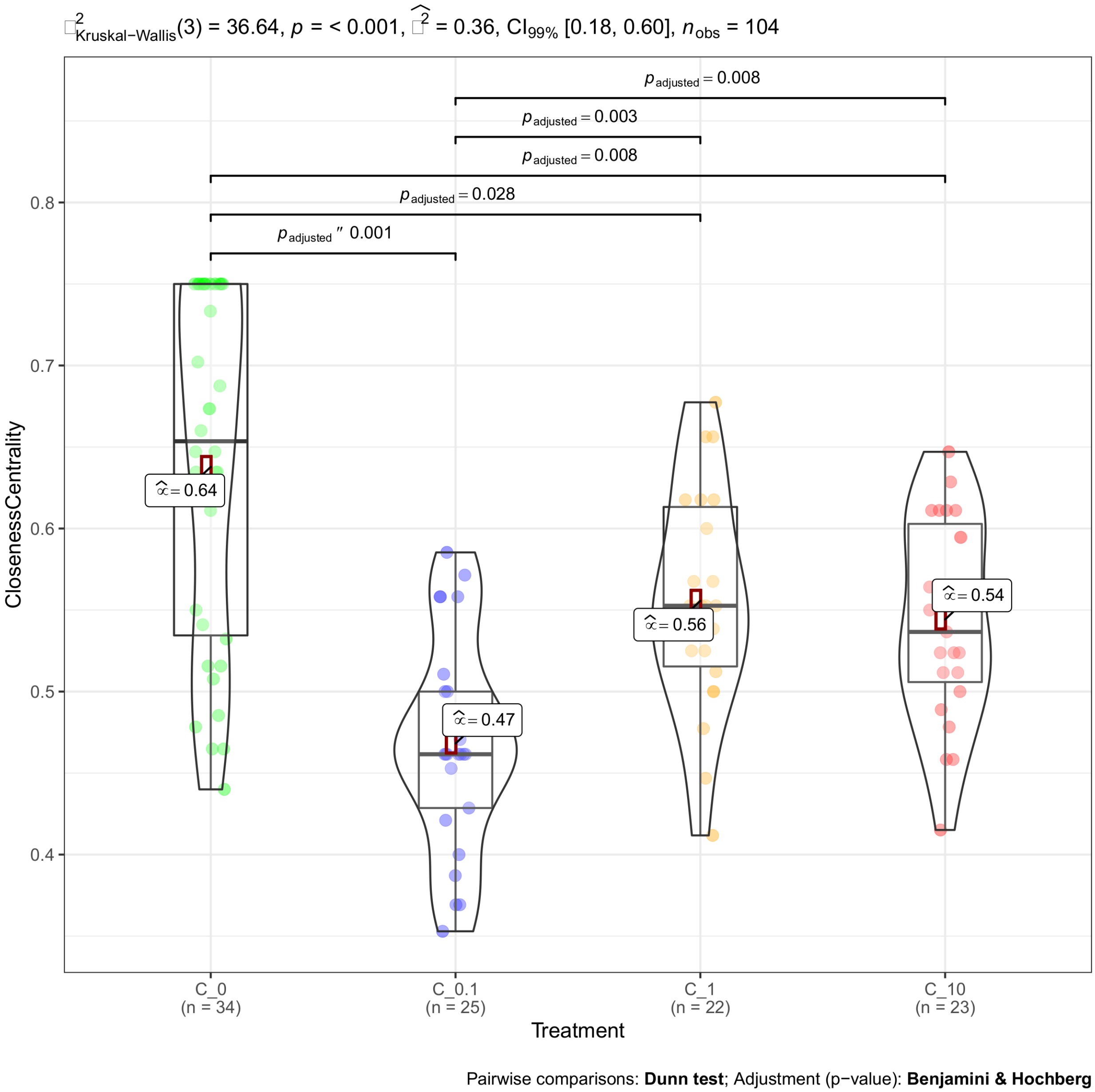

Figure S9 : Violin plot of Neighborhood Connectivity from taxa in rectum microbial networks

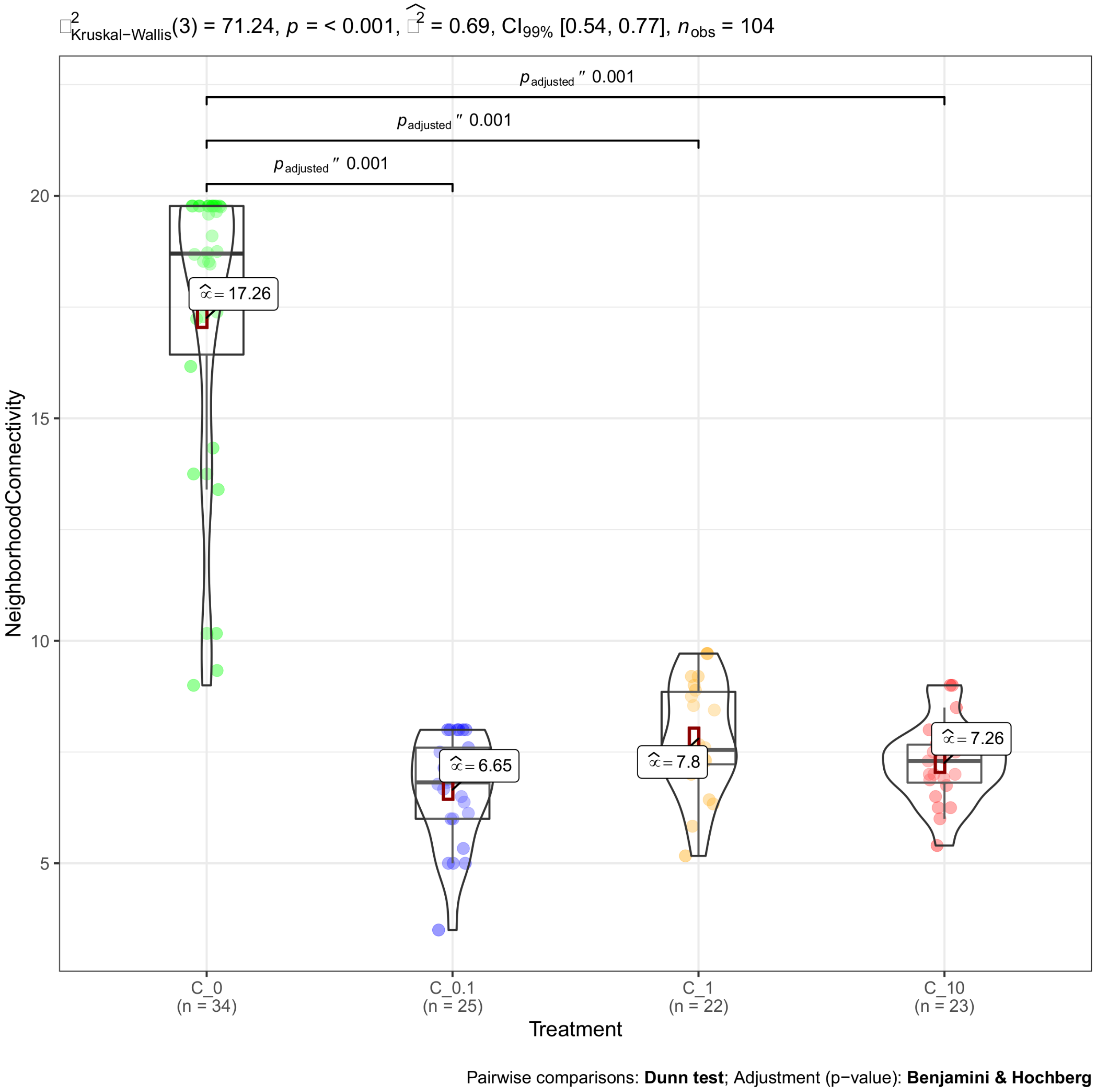

Supplement: Supplementary file 1 [file microorganisms-09-02283-s001.zip › microorganisms-1412046-supplementary.pdf]
